# Precise measurement of CRISPR genome editing outcomes through single-cell DNA sequencing

**DOI:** 10.1016/j.omtm.2025.101449

**Published:** 2025-03-14

**Authors:** Nechama Kalter, Saurabh Gulati, Michael Rosenberg, Qawer Ayaz, Joanne Nguyen, Shu Wang, Benjamin Schroeder, Chieh-Yuan Li, Ayal Hendel

**Affiliations:** 1 The Institute for Advanced Materials and Nanotechnology, The Mina and Everard Goodman Faculty of Life Sciences, Bar-Ilan University, Ramat-Gan 529002, Israel; 2 Mission Bio, 400 E Jamie Ct, Suite 100, South San Francisco, CA 94080, USA

**Keywords:** genome editing, CRISPR-Cas9, single-cell, DNA sequencing, off-target, safety, genotoxicity, cancer immunotherapy, genomic instability

## Abstract

Gene therapy for clinical applications necessitates a comprehensive, accurate, and precise measurement of gene-edited drug products. State-of-the-art pipelines for evaluating editing outcomes rely primarily on bulk sequencing approaches, which are limited to population-level assessment. Here, we leveraged Tapestri, a single-cell sequencing technology for an in-depth analysis of editing outcomes. Using this platform, we characterized the genotype of triple-edited cells simultaneously at more than 100 loci, including editing zygosity, structural variations, and cell clonality. Our findings revealed a unique editing pattern in nearly every edited cell, highlighting the importance of single-cell resolution measurement to ensure the highest safety standards.

## Graphical abstract

**Figure:**
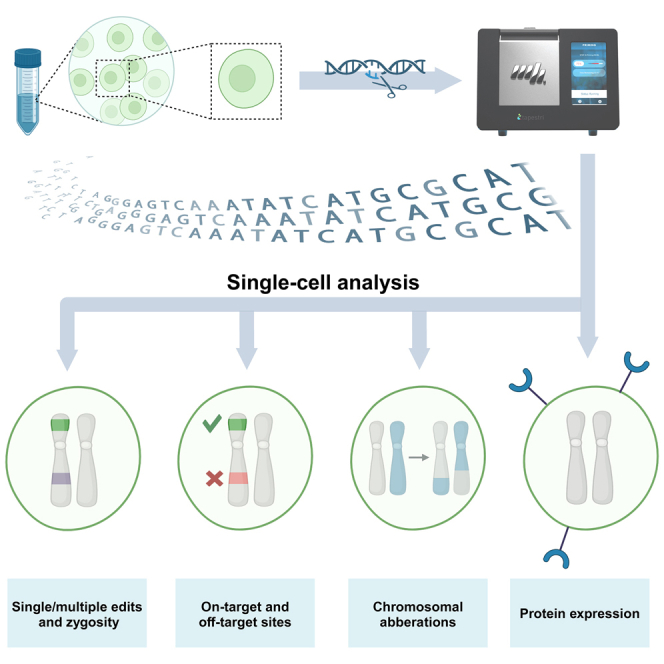

## Teaser

Kalter and colleagues use scDNA-seq to characterize CRISPR editing outcomes, highlighting its ability to assess per-cell editing activity, structural variations, and protein expression in genome-edited primary cells.

## Introduction

Genome editing (GE) through CRISPR-Cas has paved the way for a new era in treating human diseases. This technology enables the precise modification of any genomic region of interest using a custom-designed 20-bp guide RNA (gRNA) and a Cas endonuclease. The nuclease creates a DNA site-specific double-strand break (DSB), subsequently repaired by the cell’s intrinsic DNA repair mechanisms.^1^ The predominant repair pathway, non-homologous end-joining (NHEJ), involves the quick but inaccurate ligation of both DSB ends, frequently resulting in insertions and deletions (indels). In gene and cell therapy, NHEJ is leveraged to induce loss-of-function (LOF) mutations or gain-of-function mutations.^2^^,^^3^^,^^4^^,^^5^^,^^6^ Alternatively, the error-free homology-directed repair pathway, which relies on the presence of a homologous DNA fragment, mediates site-specific gene correction, and the precise introduction of large transgene sequences.^7^^,^^8^^,^^9^^,^^10^ CRISPR-Cas technology holds immense promise for gene and cell therapies, with potential applications spanning a wide array of diseases, including hereditary conditions^11^^,^^12^^,^^13^^,^^14^^,^^15^^,^^16^ and malignancies.^17^^,^^18^^,^^19^^,^^20^^,^^21^

A major safety concern in GE with targeted nucleases is off-target activity, which can result in adverse, potentially oncogenic nuclease-induced indels in unintended genomic regions.^22^ Moreover, unintended nuclease activity can induce structural variations (SVs) at on-target and/or off-target sites, including translocations, long deletions, inversions, viral-donor integration, chromosome loss, and chromothripsis.^23^^,^^24^^,^^25^^,^^26^^,^^27^ To mitigate off-target genotoxicity, rigorous monitoring of off-target activity is essential for each therapeutically intended gRNA. The identification of off-target sites can be achieved through various methodologies. Cell-based approaches, such as GUIDE-seq^28^ and DISCOVER-seq,^29^ detect off-target sites in a cellular context but have been reported to miss *bona fide* off-target sites.^30^^,^^31^ Cell-free assays, such as CHANGE-seq^32^ and SITE-Seq,^33^ are performed on naked DNA *in vitro* and are more sensitive but generate more potential off-target hits than actually occur in living cells because of the lack of cellular context. Finally, *in silico* tools, such as COSMID^34^ or CAS-OFFinder,^35^ screen for sequence homology to the protospacer sequence based on a specified Hamming distance. These pipelines are often enhanced by deep learning-based scores.^36^ Following identification, targeted bulk next-generation sequencing (NGS) is employed to amplify and quantify nuclease activity at each putative off-target site.^37^ We previously showed that multiplex targeted amplification can also capture interchromosomal and intrachromosomal translocations between pooled sites by identifying reads with primer inconsistency.^38^ Additional methods for SV detection include fluorescence *in situ* hybridization, which can detect large-scale SVs^39^; long-read sequencing technologies such as Oxford Nanopore or Pacific Biosciences' single molecule real-time sequencing^15^^,^^39^^,^^40^; and bait-and-prey methods that require a known DNA sequence, such as LAM-HTGTS^41^ and CAST-seq.^42^

While bulk NGS sequencing methods efficiently quantify adverse editing effects, they are limited to population-level analyses and lack the resolution to discern editing outcomes at the single-cell level. For example, they are unable to detect the co-occurrence of on-target edits, a critical feature when multiple genomic sites are targeted. Moreover, the bulk approaches cannot determine the zygosity of edits, which is essential when bi-allelic mutants are needed to achieve the desired phenotype.^43^ Single-cell DNA sequencing (scDNA-seq) overcomes these limitations by expanding a single-cell analysis to all genomic regions of interest. Tapestri,^44^ a scDNA-seq technology, is accompanied by a proteomics module, for a comprehensive assessment of genome-edited human cells. Ten Hacken et al.^45^ first exploited the Tapestri platform to characterize LOF mutations across multiple genomic targets in cancer cells. They introduced common oncogenic mutations across six distinct cell lines, revealing the co-occurrence and zygosity of mutations at different targets, thereby underscoring the platform’s potential for detailed characterization of mutated cells. More recently, Moshref et al.^46^ highlighted the efficacy of single-cell analysis in characterizing CRISPR-Cas9-induced effects at both on-target and off-target sites. Moshref et al. edited human CD8^+^ T cells using two gRNAs targeting the T cell receptor β locus (*TCRB*) and T cell receptor α locus (*TCRA*) genes and reported the zygosity of on-target edits, as well as the co-occurrence of edits between on-target sites and 15 putative off-target sites.

In this study, we harnessed the Tapestri technology and developed a comprehensive end-to-end assay and pipeline for the systematic evaluation of the on-target and off-target activity of CRISPR-Cas9 genome-editing products intended for therapeutic applications. By utilizing enhanced chemistry and a user-friendly, automated analysis pipeline, we generated detailed profiles of on-target and off-target effects, translocations, and proteomic outcomes. Additionally, we validated Tapestri’s performance via clonal cell lines and benchmarked it against state-of-the-art bulk analysis methods. Our approach provides per-cell and per-allele quantification of on-target and off-target editing efficiency, quantitative translocation assessment, and functional protein outcome evaluation—all within a single assay.

## Results

### Measuring CRISPR editing outcomes at a single-cell level

**Figure 1:**
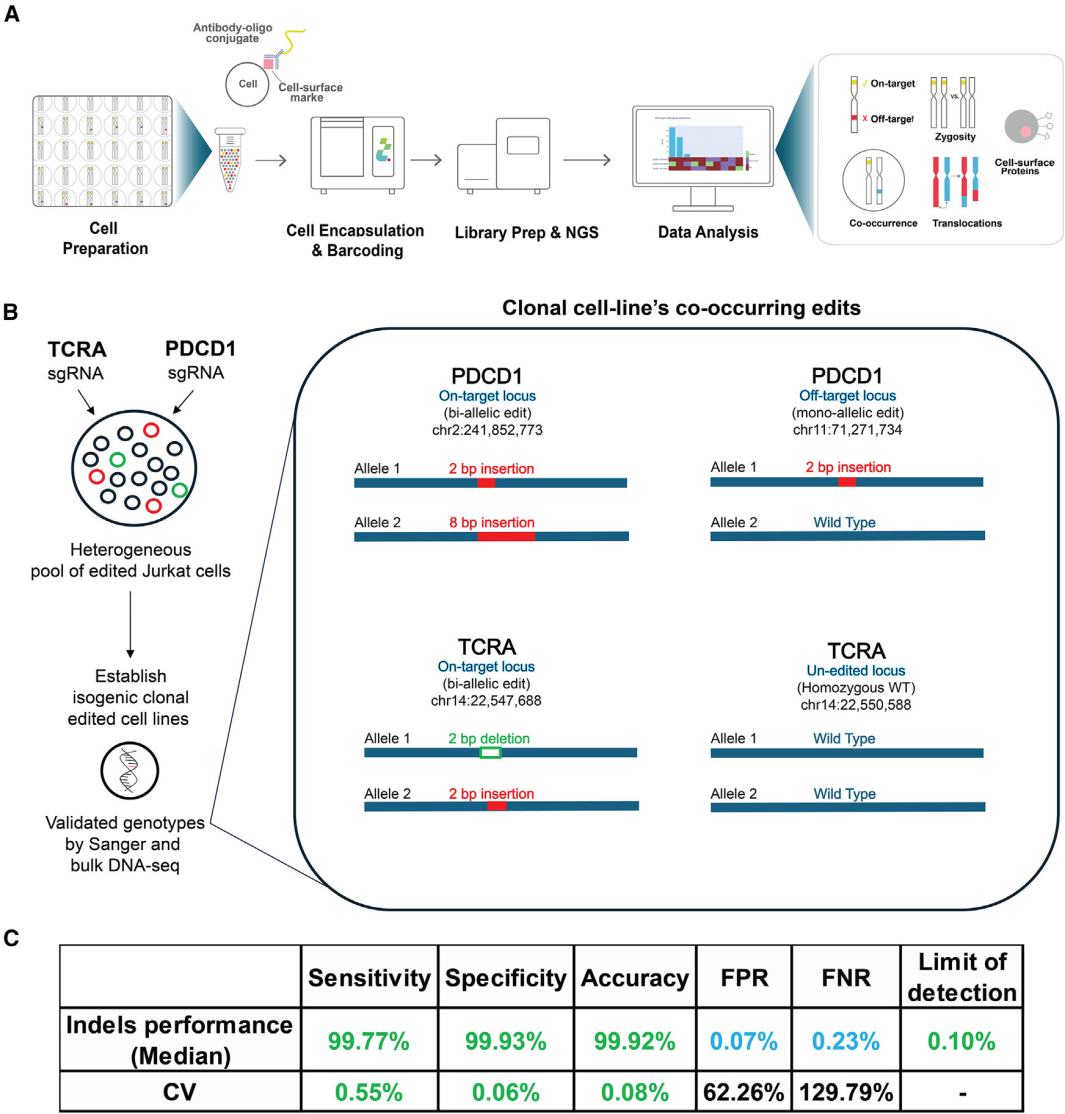
Single-cell CRISPR editing assay (A) Tapestri single-cell GE workflow. The schematic illustrates the workflow, which reports per-cell on-target and off-target edits and editing zygosity, translocations, and surface protein expression. (B) Isogenic clonal cell lines used for performance evaluation. Isogenic clones were established from Jurkat cells modified using CRISPR-Cas9 to target the PDCD1 and/or TCRA genes. Each clonal genotype was validated by bulk NGS and Sanger sequencing. The right panel shows a validated clonal culture editing outcome. (C) Performance of the Tapestri GE pipeline. The performance of the pipeline was evaluated using the isogenic clones described in (B). For each single-cell Tapestri run, only cells from a single clone were analyzed, where 100% of the sample consisted of cells with identical editing profiles. The performance of the pipeline was assessed by comparing each cell’s editing status called at each potential site, against the known reference genotype. The sensitivity, specificity, and accuracy were 99.7%, 99.93%, and 99.92%, respectively, with FPRs and FNRs of 0.07% and 0.23%, respectively, and a detection limit of 0.1%. These results are based on sequencing performed on 3 clones in triplicate (9 runs) and 1 clone in duplicate (2 runs), totaling 11 individual Tapestri runs.

To effectively characterize CRISPR-edited cell products at the single-cell level, we utilized Tapestri, a high-throughput multi-omics single-cell sequencing platform that encompasses both DNA and protein analyses. The Tapestri platform’s latest v3 chemistry employs a droplet-based, targeted resequencing method to examine specific genomic regions in parallel across tens of thousands of single cells. The process begins by encapsulating each cell in a reaction droplet, where chromatin is digested. Then, in a second droplet, a unique cell-specific barcode is attached to each DNA target through multiplex PCR (Figure 1A; materials and methods). For CRISPR-edited regions, the custom PCR panel is designed to cover both intended on-target sites and potential off-target sites, which are either predicted *in silico* or identified experimentally.^47^ Once processed through Tapestri, the samples undergo NGS and are analyzed using the automatic Tapestri GE pipeline. This pipeline provides data and comprehensive html reports on the co-occurrence and frequency of on-target and off-target editing, zygosity of the editing profile, precise editing activity per cell and per allele, and cell clonality (Figure S1; materials and methods).

To assess the performance of the Tapestri GE pipeline for detecting on-target and off-target edits, we created isogenic clonal Jurkat cell lines modified with CRISPR-Cas9 targeting programmed cell death 1 (*PDCD1*) and/or *TCRA*. Each clonal cell line’s editing profile was confirmed through bulk NGS, followed by individual processing through Tapestri, with each run including 100% of the cells from a single clone. We used a custom amplicon panel targeting both on-target and predicted off-target sites. For example, one double-edited clonal cell line displayed compound heterozygous, bi-allelic edits in *PDCD1* and *TCRA*, along with mono-allelic, heterozygous off-target edits by the *PDCD1* gRNA on chr11:71,271,734. This setup allowed us to assume that all cells from the isogenic clone contained the identified co-occurring edits (Figure 1B). Performance metrics were then calculated, focusing on zygosity at each editing site, by comparing the target’s expected editing status with the target’s editing status called by the pipeline. Metrics like sensitivity, specificity, false-positive rates (FPRs), false-negative rates, and accuracy were calculated based on classifications (true positive [TP], true negative [TN], false positive [FP], and false negative [FN]) of editing events. The pipeline performance metrics at the sample level use aggregate counts of events across all cells and targets (see materials and methods). High sensitivity (99.77%, with a % coefficient of variation [%CV] of 0.55%), specificity (99. 93%, with a %CV of 0.06%), and accuracy (99. 92%, with a %CV of 0.08%), as well as low FPR (0.07%) and FNR (0.23%) were observed for all samples with high reproducibility (Figure 1C).

### Direct measurement of single-cell-editing genotype and functional outcomes

In addition to single-cell genomic characterization, the Tapestri GE DNA + Protein pipeline reports the edit co-occurrence and zygosity for each cell, along with their corresponding quantitative surface protein expression. This is achieved by staining cells with antibody-oligo conjugates (AOCs) before processing on the Tapestri platform. Each AOC combines an antigen-specific antibody with a unique tag (barcode) oligo that yields NGS readouts for per-cell quantitative evaluation of surface antigen expression. In the context of GE, the single-cell DNA + protein workflow allows for the demultiplexing of samples by genotype (e.g., distinguishing cells from donors vs. recipients) and cell immunotyping (e.g., CD4^+^ or CD8^+^ T cells). It also enables a comprehensive analysis of each cell’s editing co-occurrence, zygosity, and confirmation of protein-level knockout (KO) in edited cells.

To demonstrate the utility of the Tapestri single-cell DNA + protein workflow, a mixture of CRISPR-Cas9 TCRA-edited Jurkat cells (heterogeneous pool) and peripheral blood mononuclear cells (PBMCs) was processed at a 55%–45% ratio. The protein panel contained 45 AOCs commonly used to evaluate hematopoietic lineages and malignancies. Successful KO of the *TCRA* gene, which encodes the TCRα subunit of the TCR complex, disrupts CD3-TCR complex formation, and diminishes the CD3 surface expression.^48^ The data show that the samples were demultiplexed through Jurkat-specific and PBMC donor-specific single-nucleotide variants, matching the intended input sample mixing ratio. Surface protein expression analysis enables the immunophenotyping of PBMC lineages such as lymphocytes (CD4^+^ and CD8^+^ T cells, B cells, and natural killer cells), monocytes, and dendritic cells. Gene editing analysis of the TCRA on-target editing site revealed that all PBMCs maintained the wild-type (WT) genotype, whereas all edited cells were Jurkat cells, as expected (Figures S2A and S2B). Specifically, in accordance with previous studies,^48^^,^^49^ unedited Jurkat cells (WT, CD3^+^) presented comparable CD3 surface expression with that of T cells in PBMCs, whereas edited Jurkat cells (mono-allelic or bi-allelic) presented diminished CD3 expression (Figure S2C).

Owing to the single-cell resolution, the editing analysis at the per-cell and per-allele levels allows for a more nuanced examination of the editing genotype and functional protein outcome. As shown in Figure S2D, there are balanced short indels across most alleles, with some cells exhibiting imbalanced indel lengths or larger indel lengths on both alleles. Whether or not the difference in indel length reflects the intrinsic properties of individual cells, such as DNA damage repair bias, or, stochastic in nature, could be further investigated. From the per-cell, per-allele analysis, each allele’s editing outcome can be classified as frameshift (FS) or non-FS (NFS) based on indel length. Indels of length multiples of three were categorized as NFS, whereas other lengths were categorized as FS. By combining this classification with per-cell editing zygosity analysis and CD3 expression levels, we confirmed that CD3 expression aligns with the expected results. Specifically, bi-allelic WT Jurkat cells exhibited the highest CD3 expression, followed by bi-allelic NFS edits. Mono-allelic FS, where the other allele may carry either an NFS edit or remain WT, and bi-allelic FS edits showed the lowest levels. The levels of CD3 expression across each category were compared pairwise using the Tukey honest significant difference range test^50^ and were all identified as significant (Figure S2E). Overall, the single-cell multi-omics workflow accurately quantifies cell-surface proteins and genome edits, enabling validation of surface protein KOs and providing insights into the nature and state of edited cells.

### Single-cell sequencing enables a comprehensive and precise evaluation of multiplex CRISPR editing efficacy in primary human cells

**Figure 2:**
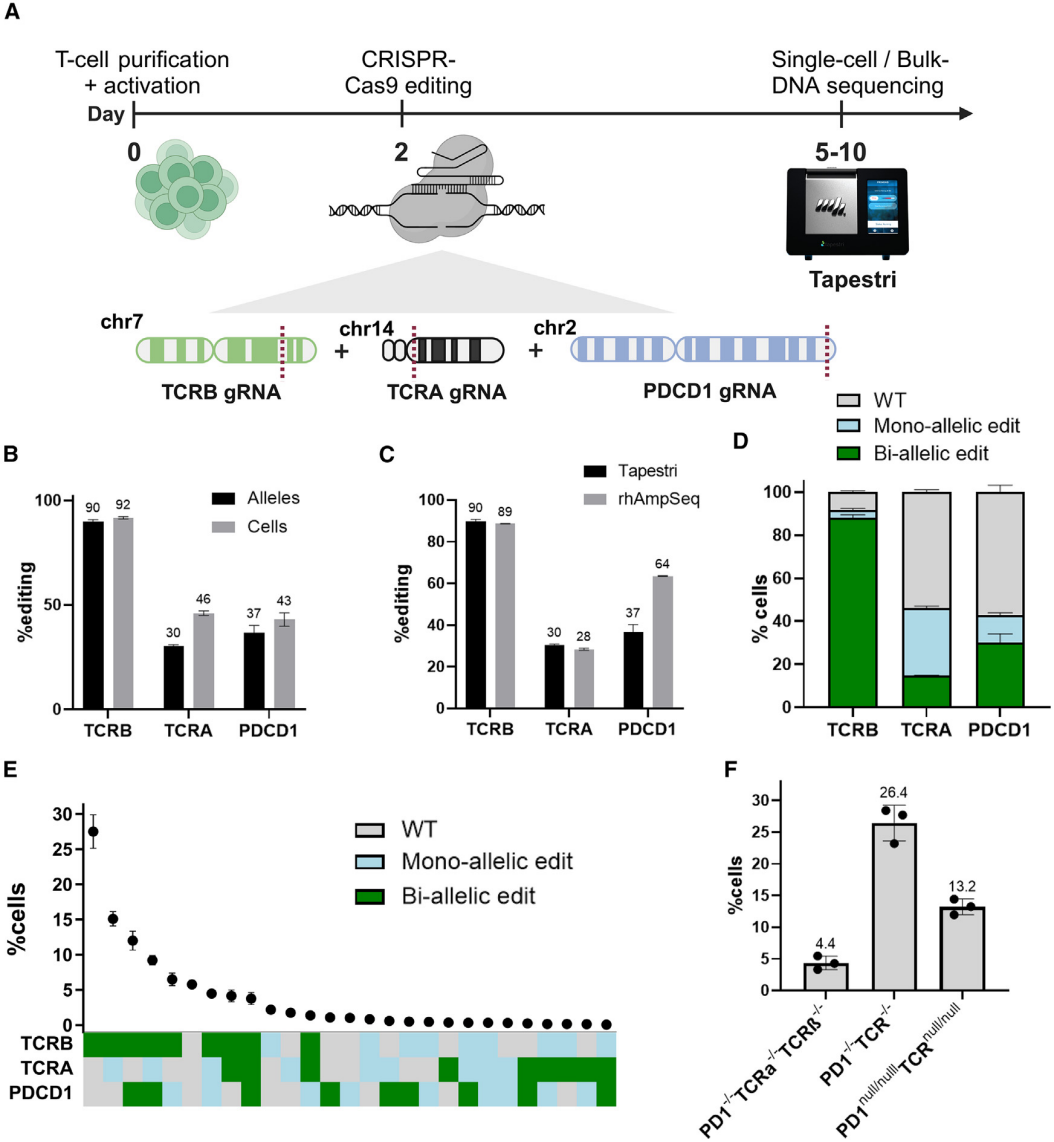
Analyzing on-target CRISPR efficiency at single-cell resolution (A) Schematic representation of the multiplex editing experiment for a triple KO of the *TCRB*, *TCRA*, and *PDCD1* genes. (B and C) Editing activity at the on-target sites as determined by Tapestri (*n* = 3, 7–10.5K cells per sample) and rhAmpSeq (*n* = 2). (B) Tapestri results at each on-target site, at the allele and cell levels (black and gray bars, respectively). (C) While editing frequencies for *TCRA* and *TCRB* loci were similar, editing activity at the *PDCD1* locus was higher when measured by rhAmpSeq (Tapestri: 37% ± 3.5%; rhAmpSeq: 63% ± 0.19%). (D–F) Additional information provided by single-cell sequencing. (D) Zygosity of on-target edits, shown as the fraction of cells with a bi-allelic edit, mono-allelic edit, or no edit at the indicated locus (green, blue, and gray bars, respectively). (E) Co-occurrence of editing events at on-target sites. (F) Fraction of the target cell population with a KO of TCR and PD1 receptors. Left bar, cells with a bi-allelic edit at all three target sites (PD1^−/−^TCRα^−/−^TCRβ^−/−^); middle bar, bi-allelic edit at the *PDCD1* locus and in at least one *TCR* gene (TCRα, TCRβ, or both) (PD1^−/−^TCR^−/−^); right bar, FS bi-allelic edit at the *PDCD1* locus and in at least one *TCR* gene (PD1^null/null^TCR^null/null^). The data are presented as mean (SD).

Following the experiments demonstrating the Tapestri performance in cell lines, we applied the single-cell sequencing GE workflow on primary T cells from two healthy donors (here referred to as D1 and D2), edited simultaneously in the *PDCD1*, *TCRA*, and *TCRB* genes, with sgRNAs identical to those used in the first clinical trial involving CRISPR-engineered T cells.^51^ Briefly, primary T cells were electroporated with a ribonucleoprotein (RNP) complex composed of Cas9 and the three sgRNAs. As a consequence of donor-to-donor variation in proliferation rates, the cells were harvested 5 days after electroporation (D1) or 10 days after electroporation (D2), to obtain a sufficient number of cells for the analysis, as described in the Methods. We then performed targeted sequencing either at the single-cell level, using the Tapestri platform, or at the population level, using the rhAmpSeq assay (see materials and methods) (Figure 2A). For Tapestri, we performed three technical replicates, each containing different cells, for each donor, along with a single unedited (WT) sample per donor. We sequenced 4,000–10,600 cells per sample, generating a total of 78–150 million reads per sample (Table S1). For rhAmpSeq, we conducted two technical replicates (using different DNA from the same cell pool) for each donor, along with two WT samples per donor. Between 0.7 and 1.6 million reads were sequenced per sample (Table S2).

While editing activity in the conventional bulk-sequencing methods is estimated by the percentage of edited DNA molecules out of the total population,^38^ in the single-cell approach, we calculated the proportion of edited cells and alleles with indels at the cut sites, observing varying editing frequencies at the cell level (D1, *TCRB*: 91.6% ± 0.57%; *TCRA*: 46% ± 1%; *PDCD1*: 43% ± 3.2%; D2, *TCRB*: 85% ± 0.6%; *TCRA*: 38% ± 1.6%; *PDCD1*: 42% ± 1.8%; mean ± SD values are indicated) and the allelic level (D1, *TCRB*: 89.9% ± 0.9%; *TCRA*: 30% ± 0.66; *PDCD1*: 37% ± 3.5%; D2, *TCRB*: 80.5% ± 0.2%; *TCRA*: 24.9% ± 1.1%; *PDCD1*: 35% ± 2.2%; mean ± SD values are indicated) (Figures 2B and S3A). We then compared the allelic-level editing rate with the indel rate determined by the rhAmpSeq assay, using Lin’s concordance correlation coefficient (CCC) analysis.^52^ For the first donor, editing frequencies for *TCRA* and *TCRB* targets exhibited an overall agreement (D1: CCC(*TCRA*) = 0.6; CCC(*TCRB*) = 0.72, D2: CCC(*TCRA*) = 0.61; and CCC(*TCRB*) = 0.72). However, for the *PDCD1* locus, editing activity measured by rhAmpSeq was higher, leading to weak agreement (CCC values: D1, 0.18; D2, 0.32) (Figures 2C and S3B). To further assess the congruence between single and bulk sequencing, we compared the distribution of indel lengths for each on-target site. We observed similar results between the assays for the *TCRA* and *TCRB* loci, while for the *PDCD1* on-target site there was an abundance of long (>40 bp) indels in the rhAmpSeq samples, particularly in D1 (Figure S4). The long deletions in the *PDCD1* locus correspond with the differences in editing activity for this donor and are likely to arise from differences in amplicon design or library preparation. Overall, our results indicate that population-level analysis using bulk sequencing is comparable with that achieved with single-cell approaches.

### Comprehensive characterization of the genotype and editing outcomes through scDNA-seq

In autosomal genes, mutations at both alleles are necessary to achieve a complete KO. Hence, we measured the heterozygosity of the edits at each edited locus. We observed different frequencies of bi-allelic edited cells for each gRNA, with those demonstrating higher editing activity showing a greater percentage of bi-allelic edits (Figures 2D and S3C). We next characterized the co-occurrence of bi-allelic or mono-allelic editing events across the different targets. Fewer than 5% of the cells harbored bi-allelic edits at all on-target sites (PD1^−/−^TCRα^−/−^TCRβ^−/−^) (D1, 4.4%; D2, 3.9%). Furthermore, the proportion of WT cells unedited at any of the targets tested (D1, 5.5%(0.5); D2, 9.6%(1.5)) was lower than the proportion of WT cells calculated for each amplicon separately (Figures 2E, 2F, S3E, and S3F). Assuming independence among the on-target sites, the probability of any combination of mono-allelic or bi-allelic edits at each site in the multiplex-edited cells can be estimated by multiplying the frequencies of the respective edits at each target. For instance, the likelihood of achieving bi-allelic edits across all on-target sites can be calculated as p(TCRA_bi-allelic) × p(TCRB_bi-allelic) × p(PDCD1_bi-allelic). Comparison of the expected (Exp) results per each editing combination shown in Figure 2E revealed a strong positive correlation with the observed (Obs) data (Pearson r^2^ across three replicates; D1: 0.96, 0.97, 0.97; D2: 0.97, 0.94, 0.98) (Figure S5), indicating that editing events at the on-target sites are independent, and no positive or negative selection for specific subpopulations occurred in this specific dataset.

We further evaluated whether co-occurrences of editing combinations can be predicted using the bulk-sequencing data. As bulk data does not provide information about the zygosity of edits (mono-allelic or bi-allelic), we focused on evaluating the likelihood of any target to be edited or unedited. The expected probability for each combination of edited or unedited targets was calculated as the product of the individual probabilities derived from rhAmpSeq observed (Obs) results, represented as Exp(TCRA, TCRB, PDCD1) = Obs(TCRA) × Obs(TCRB) × Obs(PDCD1). For the first donor, observed and expected values were moderately correlated (Pearson r^2^ across three replicates = 0.47, 0.50, 0.53), whereas a stronger correlation was noted for the second donor (Pearson r^2^ across three replicates = 0.72, 0.78, 0.85) (Figure S6). Overall, our findings suggest that, in the absence of target interdependence and selective enrichment of specific cells, the proportions of subpopulations with diverse editing outcomes can be inferred to some extent from bulk data.

Nonetheless, a simple binary classification of edited versus unedited, or even bi-allelic versus mono-allelic edits, is often insufficient, as not all editing outcomes necessarily result in the desired phenotype (e.g., protein KO). Therefore, a more detailed analysis that examines the specific edits (indels) in each cell is required. In the context of the genome-editing design tested in this manuscript, the goal is to introduce KOs to either the *TCRA* or *TCRB* genes, encoding the TCRα and TCRβ subunits of the TCR, respectively, as well as in the *PDCD1* gene, which encodes the PD-1 receptor. Therefore, the desired cell product would contain a bi-allelic null mutation (defined here as an FS mutation or a >11 bp indel) in at least one *TCR* gene, along with a bi-allelic null mutation in the *PDCD1* locus (PD1^null/null^TCR^null/null^) (Figure S3D). While the frequencies of cells with any bi-allelic indels at the *PDCD1* and *TCR* loci (PD1^−/−^TCR^−/−^) were high (D1, 26.4%; D2, 24.1%), only one-half of these cells (D1, 13.2%; D2, 13.1%) contained the desired double-KO null mutation (Figures 2F and S3F). Albeit, given the allelic exclusion phenomenon following VDJ recombination, it should be anticipated that a certain percentage of the cells single edited on *TCRA* or *TCRB* can lose expression of the TCR, which, alongside *PDCD1* biallelic KO, would yield the desired outcome.^53^ Taken together, our data suggest that population-level analysis falls short in accurately assessing the efficiency of editing outcomes, and that comprehensive single-cell methodology is indispensable, particularly for complex products targeting multiple loci.

### Multiplex GE in primary human cells generates heterogeneous editing outcomes at on- and off-target sites

Beyond evaluating efficiency, it is essential to characterize the off-target profiles of each genetically modified product to mitigate the risk of unintended genotoxicity. Since there is no known algorithm or assay that can pinpoint the *bona fide* off-target sites out of an ensemble of putative off-target sites, experimental validation of off-target activity on multiple genomic sites simultaneously is critical to mitigate any potential risk. Therefore, we subsequently evaluated the specificity of the TCRA, TCRB, and PDCD1 gRNAs at single-cell resolution using Tapestri. To identify potential off-target sites, we conducted GUIDE-seq experiments in a HEK293 cell line with a stable Cas9 expression (HEK-Cas9) for each gRNA individually. We then performed targeted amplification (using rhAmpSeq) on the top GUIDE-seq sites for each gRNA using the HEK-Cas9 system. Since off-target activity is cell type dependent,^31^^,^^37^ we applied rhAmpSeq to triple-edited primary T cells (data not shown). Based on these experiments, we designed a custom 115-plex panel encompassing both on-target sites and top-ranking off-target sites for the Tapestri and rhAmpSeq assays (Tables S3 and S4, respectively). This panel was then applied to triple-edited cells from two primary human T cell donors (D1 and D2), and the resulting data were analyzed using the Tapestri GE pipeline.

**Figure 3:**
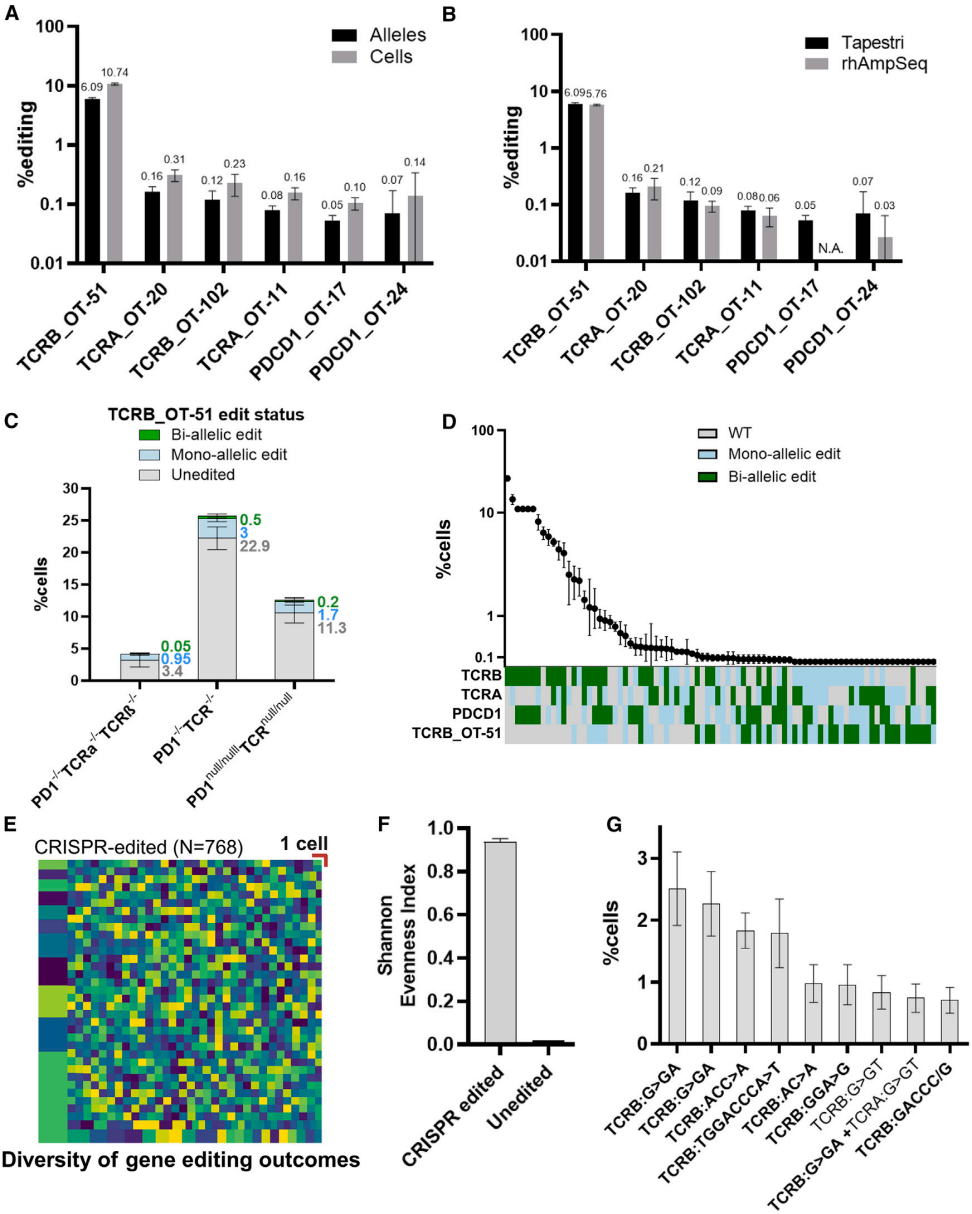
Analyzing off-target CRISPR activity at single-cell resolution (A and B) Off-target activity as determined by Tapestri (*n* = 3, 7–10.5K cells per sample) and rhAmpSeq (*n* = 2). Sites with >0.1% editing in one of the methods are presented. (A) Tapestri-measured off-target editing activity at the allele and cell levels (black and gray bars, respectively). (B) Comparison between the Tapestri (black bars) and rhAmpSeq results (gray bars). (C and D) Co-occurrence of the most active off-target site (TCRB_OT-51) with the desired on-target editing outcomes. (C) Fraction of cells with an off-target activity in TCRB_OT-51 out of the target cells shown in Figure 2F. (D) Co-occurrence of editing events at the on-target sites and TCRB_OT-51. (E) Treemap visualization showing the diverse editing outcomes, for a one replicate of edited cells. Each rectangle represents the fraction of cells with a specific combination of editing outcomes (different type of indels) for each target, in each allele. The size of each rectangle corresponds to the relative abundance of that cell population. For reference, one of the smallest rectangles, representing a single cell, is marked with a red scale in the figure, while larger rectangles indicate proportionally higher cell numbers. (F) Shannon Evenness Index was used to assess the diversity of editing outcomes in the samples. The left bar represents the average Shannon index for CRISPR-edited samples, while the right bar shows the value for the WT sample. The treated samples displayed a high diversity of editing outcomes, as indicated by an elevated average Shannon evenness index of 0.91 ± 0.01. (G) The most frequent editing outcomes observed in the edited population, out of the clones shown in Figure 2E, with the specific indels observed for that clone detailed. Bi-allelic indels are indicated in bold, and mono-allelic edits are shown in Roman font. The data are presented as mean (SD).

Among the tested off-target sites, six exhibited editing activity above the limit of detection in at least one donor (0.1% of cells), with one prominent off-target site, TCRB_OT-51, consistently displaying high editing frequencies in both donors (Figures 3A and S7A). We then amplified the same genomic DNA from D1 and D2 using bulk sequencing, with a rhAmpSeq panel designed for the same targets as the single-cell panel. The bulk-sequencing results were consistent with the single-cell data, with the exception of the PDCD1_OT-17 site, which was amplified solely by the single-cell panel (Figures 3B and S7B). We next assessed the co-occurrence of editing events between on- and off-target sites, focusing on the highly active TCRB_OT-51 off-target site (Figures 3C and S7C). We analyzed the desired edited populations (PD1^−/−^TCRα^−/−^TCRβ^−/−^, PD1^−/−^TCR^−/−^, and PD1^null/null^TCR^null/null^), while also considering off-target effects. Among the initial numbers shown in Figure 2F, a subset of cells contained mono-allelic or bi-allelic edits at the TCRB_OT-51 site, resulting in a 0.5%–3% decrease in the final, off-target-free, target KO cell population (Figures 3C, 3D, S7C, and S7D).

Next, we characterized the specific indel patterns at both on- and off-target sites for each cell, mapping the post-editing genotype for every cell at each amplified site, focusing on the on-target sites and TCRB_OT-51. We analyzed the clonality of the sample, where each clone represents a specific indel type, at each allele at each mentioned target. We observed a high diversity of editing outcomes among edited cells, with nearly every cell displaying a unique editing profile (Figures 3E and S7E). Correspondingly, average Shannon Evenness Index (*J*) values for the edited samples were high (*J*(D1): 0.91 ± 0.01; *J*(D2): 0.9 ± 0.005), hinting on high richness of editing outcomes in the population. For the WT sample, where only unedited cells are evident, *J* values were below 0.01 (Figures 3F and S7F). Consistent with the population-level editing results, the most frequently observed genotypes displayed bi-allelic or mono-allelic indels at the *TCRB* locus, with no indels detected at other target sites (Figures 3G and S7G). However, even within this subset, fewer than 3% of cells shared identical editing outcomes, further underscoring the degree of heterogeneity within the edited samples.

### Single-cell sequencing supports quantitative translocation detection

**Figure 4:**
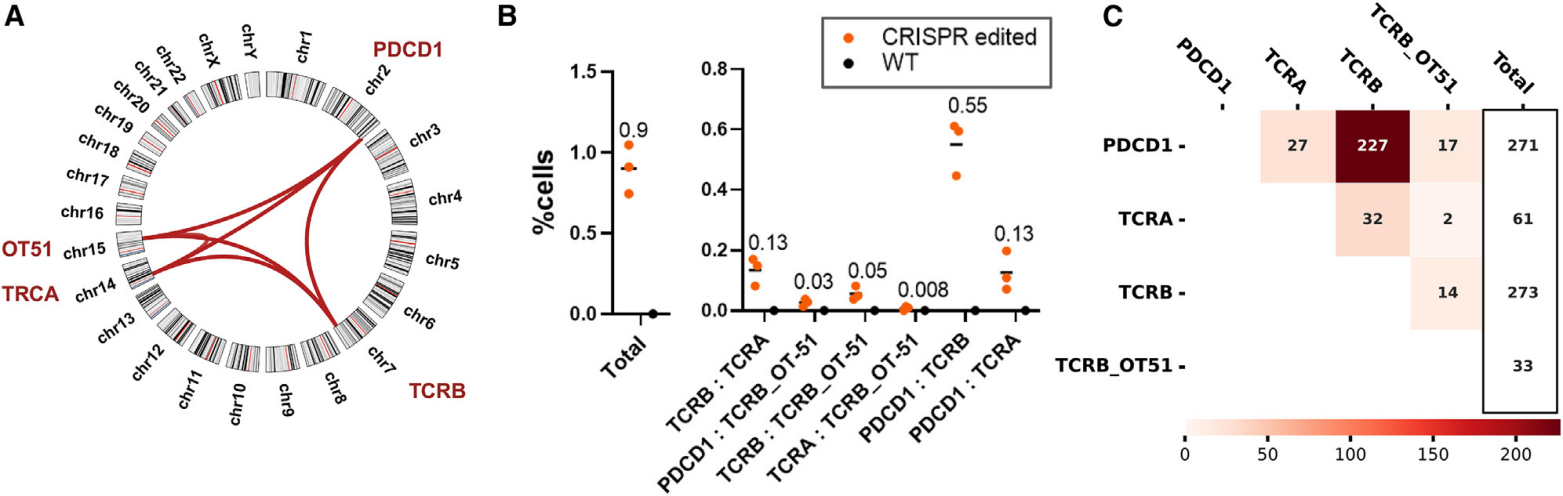
Using single-cell sequencing for translocation detection in editing experiments (A) Circos plot showing translocations identified within the multiplex-edited population using single-cell sequencing. (B) Overall percentage of cells harboring translocations, as identified through single-cell sequencing (left), and distribution of specific translocation events within the cell population (right) (n = 3, 7–10.5K cells per sample). Mean values are presented. (C) Translocations validated by bulk NGS. Numbers indicate read count for each translocation event, in both replicates combined.

Another adverse outcome of CRISPR-induced DSBs at on-target or off-target sites is the formation of SVs, such as translocations, deletions, and inversions, which pose significant risks of oncogenic mutations. Single-cell sequencing facilitates unbiased, quantitative SV detection, particularly of translocation events, by identifying chimeric reads (amplicons from primers targeting different loci and harboring both respective sequences) (see materials and methods). Additionally, single-cell resolution allows the assessment of co-occurring translocations and translocation/indel events. In the multiplex-edited cells, we identified translocations between the three on-target sites, in all possible combinations, as reported previously.^26^ Furthermore, we identified novel translocations between the TCRB_OT-51 off-target site and each of the three on-target sites (*TCRB:TCRB_OT-*51, *TCRA:TCRB_OT-51*, and *PDCD1:TCRB_OT-*51), in both donors, in frequencies below 0.1%. These translocations were not identified in previous publications exploring chromosomal aberrations with the same gRNAs.^25^^,^^26^ Notably, the *TCRA:TCRB_OT-51* translocation was observed with a frequency as low as 1 in 25,000 cells, demonstrating the sensitivity of the single-cell approach in detecting SVs. Overall, approximately 1% of the edited cells harbored translocations (D1: 0.9% ± 0.15%, *n* = 3; D2: 1.15% ± 0.25%, *n* = 3; mean ± SD values are presented) (Figures 4A, 4B, S8A, and S8B).

We previously demonstrated that qualitative translocation detection at the population level can be effectively performed using multiplex PCR followed by targeted amplicon sequencing, such as rhAmpSeq. Bulk sequencing has been shown to detect translocations present in as few as 0.016% of cells.^38^ For the first donor, the rhAmpSeq data identified all translocations captured by Tapestri, including the low-frequency, previously unreported TCRB_OT-51 translocations. However, for the second donor, rhAmpSeq detected only translocations occurring between the on-target sites, suggesting the potential superiority of the single-cell method in detecting low-incidence translocations (Figures 4C and S8C).

### Integrated scDNA-seq and proteomic analyses validate functional KO in edited primary cells

In addition to providing genotypic information through scDNA-seq, the Tapestri technology allows the integration of a custom proteomic panel to quantitatively characterize cell-surface antigens within the same sequenced cells. For genome-editing studies, such integrated analysis facilitates the linkage between genotype and phenotype, validates functional KOs of target proteins, and elucidates the specific editing outcomes required to achieve the desired phenotype. To explore the correlation between editing outcomes achieved in the *TCRA*-*TCRB*-*PDCD1* design and their effects at the protein level, we edited primary T cells from an additional donor (here denoted as D3), edited using an RNP of Cas9 and the three sgRNAs similar to the previous donors. To elucidate dynamics of protein KO, samples were collected at multiple timepoints (6 h, 24 h, 3 days, 7 days, and 12 days post editing) and compared with an unedited control sample. In each time point, cells were sequenced and stained using a 47-plex multi-omics panel.

**Figure 5:**
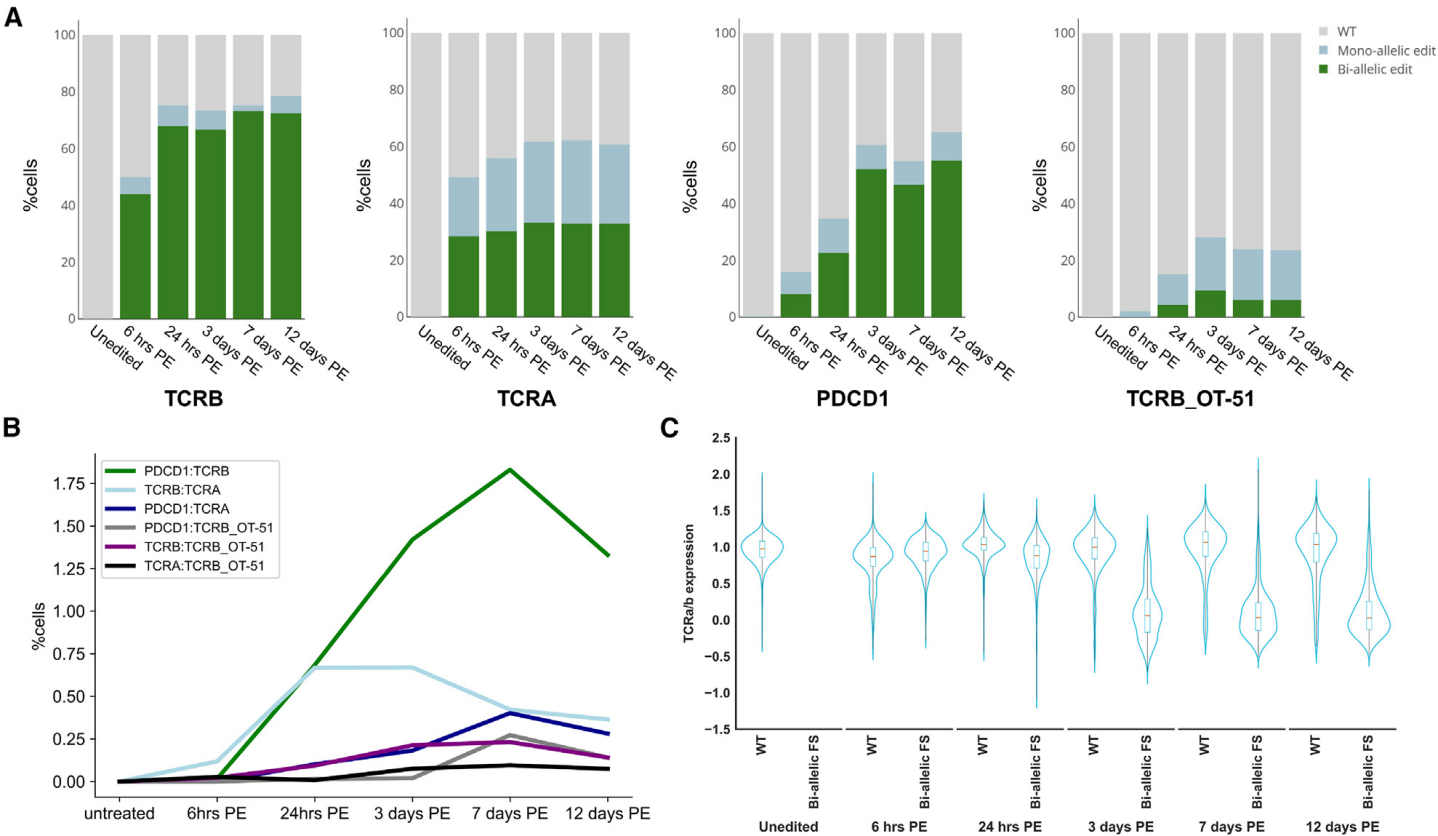
scDNA-seq combined with proteomics validates functional KO of TCRα/β in cells with edits at the TCR loci (A) Zygosity of editing in D3 across timepoints, shown for the *TCRB*, *TCRA*, and *PDCD1* on-target sites, and TCRB_OT-51. The percentage of total cells with indels increased from 6 h to 3 days post editing, with a stable percentage beyond this time point. Percentage of cells with a bi-allelic indels is shown in green, mono-allelic edits in blue, and WT portion in gray. (B) Rate of translocation across timepoints in D3. Each line represents a distinct translocation. (C) Violin plots depicting the TCRα/β cell surface expression for the timepoints tested. For each time point, cells are categorized using the following definitions: WT (cells with no *TCRA* or *TCRB* editing), and bi-allelic FS (cells harboring a bi-allelic FS edit on either *TCRA* or *TCRB*). Boxplots within each violin represent the distribution of cells, with the horizontal red lines representing the median values. PE, post editing.

Editing rates at the different target sites, measured by the frequency of indels, increased progressively post editing, with peak editing observed between 24 h and 3 days, reflecting active nuclease activity during these early time points. Editing rates remained stable through the later time points, indicating the persistence of edits in the population. The total number of cells containing indels on day 12 were similar to those observed for D1 and D2, which were sampled on days 5 and 10 post electroporation (*TCRB*, 94%; *TCRA*, 60.6%; *PDCD1*, 65%), while off-target activity in TCRB_OT-51 was higher in D3 (23.5%) (Figure 5A). The percentage of cells expressing each translocation increased from 6 h to 7 days post editing. After this peak, however, the frequency of translocations gradually decreased over time, suggesting a potential loss of fitness for those cells due to selective pressures. The *TCRB:TCRA* and *PDCD1:TCRB* translocations, which were most prevalent in D1 and D2, were also observed at higher frequencies in D3-edited cells compared with unedited controls, with *TCRB:TCRA* translocations reaching 0.3% and *PDCD1:TCRB* translocations reaching 1.3% at 12 days post editing. Translocations involving TCRB_OT-51 were less prevalent but still detectable across time points, with a total of 0.3% of cells containing any TCRB_OT-51 translocation 12 days post editing (Figure 5B).

We next sought to determine how the observed genotypes are mirrored at the proteomic level. We hypothesized that cells harboring a bi-allelic FS indel in either *TCRA* or *TCRB* loci would lose membrane-bound TCR. Consequently, we anticipated a downregulation of the associated CD3 subunits in these cells. For each time point, we have grouped the WT cells, which do not contain any edit in *TCRA* and *TCRB*, and the bi-allelic FS cells, with a bi-allelic FS mutation in either *TCRA* or *TCRB* on-target sites. For the WT subset, the overall distribution of TCRα/β expression was similar across timepoints and was similar to those of the unedited population. In contrast, in the bi-allelic FS cells, TCRα/β expression steadily decreased from 6 h to 3 days (Figures 5C and S10A), with more than 80% of bi-allelic FS cells showing no expression of the TCRα/β and CD3 protein between day 3 and day 12 (Figures S10B and S10C). These findings are consistent with our observations in Jurkat cells, where mono-allelic cells were also shown to lose TCR expression. Our results strongly support that genotyping cells at the single-cell level correlates with the functional disruption of target proteins. Altogether, when combined with proteomics, the Tapestri platform offers a comprehensive, end-to-end solution for genotype and proteomic evaluation of edited cells.

## Discussion

Precise measurement of editing outcomes in gene and cell therapy experiments is essential for ensuring the fidelity and efficacy of therapeutic products. In this study, we present an scDNA-seq approach that employs the Tapestri technology to provide a comprehensive, end-to-end pipeline for evaluating the efficacy, safety, and functionality of engineered cells. Utilizing Tapestri and the GE pipeline, we simultaneously measured nuclease activity at both on-target sites and numerous off-target sites with per-cell, per-allele precision. Additionally, we assessed the zygosity of edits and the co-occurrence of events at the indel level per allele. Our approach also enabled the characterization of translocations, including the identification of a novel, previously unreported translocation.

The current gold standard for measuring and quantifying on-target efficiency and off-target activity relies on bulk sequencing methods. We demonstrated using dozens of on-target and off-target sites that population-level analysis is largely consistent between methods. For the majority of both on-target and off-target sites tested in this study, we observed high concordance between the two assays. However, for the *PDCD1* on-target site, we noted differences in editing activity between the rhAmpSeq and Tapestri results. Specifically, the Tapestri amplicon was shorter than the rhAmpSeq amplicon (209 bp vs. 246 bp, respectively). The *PDCD1* on-target site contains microhomology regions near the cut sites, making it susceptible to alternative-NHEJ-mediated long deletions. These long deletions likely caused shortened amplicon reads in Tapestri, which impacted amplification efficiency, leading to a reduced number of reads per cell and an under-representation of the deletions in the final sequencing data. Optimization of amplicon designs could improve the retrieval of additional editing outcomes. For clinical applications, we recommend validating the assay design of each target to ensure accurate estimation of the genotype in engineered cells.

While bulk-sequencing methods are both efficient and cost effective, they are limited in their ability to capture the full spectrum of editing outcomes. Specifically, they cannot accurately ascertain the co-occurrence of on-target edits, a crucial feature when multiple targets are edited simultaneously, as is often the case in immunotherapy. Moreover, bulk sequencing-based methods quantify the proportion of edited DNA molecules relative to the total DNA, a method that, while relatively accurate, fails to precisely capture the number of edited alleles and cells. In experiments requiring a large number of input cells, such as in sickle cell disease, precise assessment is crucial to avoid insufficient input. Population-level analyses also cannot determine the zygosity at each allele, a feature that is essential when modeling recessive diseases, or when both copies should be mutated to replace the endogenous gene expression completely with transgenic expression, as in the case of the TCR. Furthermore, the specific combination of editing outcomes at various on-target and off-target sites within each cell is crucial for determining its functionality and genotoxicity profile. In immunotherapy design, for instance, achieving FS or nonsense mutations at all on-target sites while avoiding potentially harmful off-target mutations is essential. Inaccurate measurements can result in a lower yield of the desired cell product than anticipated. Functional assays, such as flow cytometry and cytotoxicity studies, assess the functional outcomes of the cells but do not link genotype to phenotype. Single-cell RNA sequencing (scRNA-seq) can address these limitations by offering single-cell resolution editing frequency quantification and chromosomal aberration detection.^25^^,^^26^^,^^51^ Nevertheless, scRNA-seq is restricted to coding regions. In contrast, the Tapestri scDNA-seq technology provides a comprehensive solution by enabling accurate genotype profiling alongside functional outcomes. By designing a custom panel of hundreds of on- and off-target sites across the genome, together with a tailored proteomic profile, the Tapestri single-cell platform delivers an end-to-end solution for creating safe, efficient, and functional genome-engineered cells.

Our analysis revealed a distinct distribution of cells, each characterized by a unique editing profile comprising a combination of various indel types at different alleles and sites. This finding underscores the inherent heterogeneity of the editing process, resulting in a multitude of new genomes, rather than a uniform cell population. Hence, a single-cell approach is crucial for applications requiring high fidelity and specificity, such as immunotherapy and the correction of genetic disorders, where the accurate characterization of each edited cell is paramount to the success of the treatment. Despite the observed heterogeneity post-editing, the potential for clonal expansion of mutative clones within the edited population during expansion or after transplantation poses a significant genotoxicity risk. Longitudinal studies leveraging Tapestri’s capabilities could provide deeper insights into the dynamics of clonality, enabling the detection and mitigation of clonal expansion due to adverse off-target effects. Additionally, continuous monitoring ensures that the engineered target cell population remains stable over time, maintaining its efficacy and safety.

We also demonstrated Tapestri as a powerful tool for measuring adverse SVs resulting from the Cas nuclease activity. Previously reported bait-prey methods for translocation detection, such as HTGTS or CAST-seq,^41^^,^^42^ rely on a predefined set of bait sequences, typically corresponding with the on-target site. Consequently, these assays can only detect SVs involving the bait sequence. However, they offer the advantage of identifying translocations between the bait locus and any other genomic locus, without being restricted to predefined amplicon-pooled sites. Stadtmauer et al.^51^ designed TaqMan probes specifically for the translocations between on-target sites, thus precluding the detection of the translocation events involving off-target sites. In contrast, our analysis revealed translocations not only between on-target sites but also between on-target and off-target sites. Notably, we identified a novel translocation between the TCRB_OT-51 off-target site and each of the on-target sites. Although this off-target site, located within the intron of the zinc finger protein 609 (*ZNF609*) transcriptional regulator, has previously been reported by Stadtmauer et al.^51^ as a putative off-target site, the authors did not employ a method that would lead to the detection of translocation events tied to this site. In contrast, Tapestri expands the identification of translocations and long deletions to on-target and off-target sites within all sites included in the panel. Nevertheless, the amplicon-based SV detection approach is restricted to sites within the multiplexed amplicon. While in this manuscript we focused on the detection of translocations, the technology also has the potential to detect another adverse effect, including long deletions or other types of copy number variations. Recent work demonstrated that scDNA-seq can be used to identify aneuploidy present in tumors.^54^ Tapestri can potentially also identify long deletions or other types of copy number variations by analyzing changes in read coverage across adjacent amplicons. This hypothesis will be validated in future studies.

As a targeted sequencing approach, Tapestri scDNA-seq technology is most efficiently utilized when accompanied by a preceding off-target detection or prediction step to design a tailored DNA sequencing panel. This process should be factored into the planning of experiments utilizing this technology. Additionally, the current Tapestri droplet chemistry is optimized for an upper limit of 1,000 amplicons for a single panel, hence limiting the number of sites per experiment that can be analyzed for single-cell context. Factoring in the cost of sequencing, this technology is optimal for analyzing approximately 1,000 targets of interest in thousands of cells in parallel, and less ideal for broad-range genome screening purposes. When compared with other single-cell technologies, Tapestri offers comparable costs per sample and similar input cell requirements, as low as a few thousand cells. Although single-cell technologies are more expensive than bulk studies, Tapestri’s targeted sequencing approach, as opposed to whole-genome sequencing, significantly reduces overall costs and offers a cost-efficient method providing high-throughput single-cell analysis.

Moshref et al.^46^ previously used scDNA-seq to identify translocations between on-target sites of two different gRNAs, targeting *TCRA* and *TCRB* loci in edited primary cells, along with a 14-site off-target panel. Their study demonstrated the feasibility of scDNA-seq for measuring the co-occurrence and zygosity of editing events and leveraged its proteomic capabilities to show that different subpopulations of primary cells exhibited similar editing profiles. Additionally, translocations between on-target sites were identified and validated using digital droplet PCR. While this work effectively highlights the potential and versatility of the technology, our study aimed to thoroughly evaluate its performance using clonal cell lines and to demonstrate its capabilities and advantages through direct comparison with gold standard bulk-sequencing assays. The gRNA sequences used for disrupting the *TCR* genes in primary cells differ between the two studies; we utilized clinically relevant guides as described by Stadtmauer et al.^51^ and included a broad 115-plex panel. Moreover, in our manuscript, we utilized improved V3 chemistry, which provides better uniform amplicon representations, increases cell capture rate, and implemented an automated analysis pipeline to ensure accurate and reproducible results. Finally, we applied the multi-omics panel to perform an in-depth evaluation of the functional outcomes of different editing types in a longitudinal study, highlighting the practical analytical potential of the technology as a holistic and integrative approach to assessing the interplay between genotype and phenotype in genome-engineered cells.

### Conclusions

Our study highlights the transformative potential of scDNA-seq technology in enhancing the precision of GE products. By integrating single-cell resolution with a comprehensive analysis of both on-target and off-target effects, the single-cell approach refines our ability to assess the efficacy and safety of genome-engineered therapies, by ensuring a more accurate characterization of each edited cell, paving the way for more reliable and effective gene and cell therapy drugs.

## Materials and methods

### Single-cell DNA and protein sample preparation and sequencing

Mission Bio’s Tapestri is a targeted single-cell DNA + protein sequencing platform that allows for the interrogation of genomic regions of interest and quantitative analysis of surface protein expression in thousands of cells in parallel by encapsulating and barcoding single cells and then employing bulk NGS, after which, single-cell barcodes are deconvoluted. Detailed instructions for using Tapestri’s single-cell sequencing, including the required materials, equipment, and workflow, can be found in Mission Bio’s Tapestri V3 User Guide (for DNA-only workflow) and the Tapestri DNA and Protein V3 User Guide (for combined DNA and protein workflow). The Tapestri V3 chemistry is optimized for a stable, uniform droplet PCR amplification and efficient cell capture, essential for high-throughput single-cell analysis. Optimization enables the identification of rare cells in heterogeneous populations and low-frequency events associated with gene editing (see Tapestri DNA sequencing v3 user guide). To capture editing events on on-target and predicted off-target sites a targeted sequencing panel was designed covering said targets, this panel had amplicons ranging from 190 bp to 296 bp in length.

The single-cell sequencing process follows a two-step microfluidics workflow that includes cell encapsulation, barcoding, multiplex emulsion PCR, and NGS library preparation. In the DNA plus protein workflow, cells are stained with either 45 unique primary lineage AOCs (TotalSeq-D Heme Oncology Cocktail, BioLegend) (donors 1 and 2), or with a panel with CD279 and TCR α/β AOCs spiked-in on top of the 45-plex panel (donor 3), each carrying a unique barcode for specific antibodies before loading onto Tapestri. During cell encapsulation, individual cells are quickly isolated with a lysis buffer in droplets. These droplets are incubated at 50°C to release DNA from chromatin, allowing for uniform genome examination. After lysis, in the barcoding step, droplets containing cell lysates are merged with cell barcode beads and PCR components to form barcoding droplets. These droplets undergo PCR cycles to enable targeted genomic amplification within barcoded individual cells. The emulsion droplets are then disrupted, and the amplified products are pooled for standard NGS library preparation. Sequencing was performed in a 2 × 150 bp paired-end format on the Illumina NextSeq 550 platform, generating 75–150 million mapped reads per sample (see Table S1).

### Isogenic CRISPR-edited clonal cell lines

CRISPR-Cas9-edited cell lines (Jurkat) with guides targeting *PDCD1* (sgRNA: GCAGUUGUGUGACACGGAAG; cut location chr2:241,852,773) and *TCRA* (sgRNA: ACAAAACUGUGCUAGACAUG; cut location chr14:22,547,658) were generated by Synthego. The heterozygous edited cells were isolated as single cells in tissue culture plates for clonal expansion. The co-occurring editing events and genotypes of individual isogenic cell lines were identified through Sanger sequencing and bulk NGS using PCR primers flanking the edited loci (Table 1).

**Table 1:** PCR primers used for bulk NGS and Sanger sequencing in isogenic CRISPR-edited clonal cell lines

Gene	Method	Primer type	Sequence
PDCD1	Sanger	forward	CTACGACCCTGGAGCTCCT
PDCD1	Sanger	reverse	CTGCTCGTGGTGACCGAAG
PDCD1	bulk NGS	forward	CACCTGTCACCCTGAGCTCT
PDCD1	bulk NGS	reverse	CCAGCAACCAGACGGACAA
TCRA	Sanger	forward	ACCCTGATCCTCTTGTCCCA
TCRA	Sanger	reverse	AAAGAGGGTTTTGGTGGCAA
TCRA	bulk NGS	forward	TGAGATCATGTCCTAACCCTGA
TCRA	bulk NGS	reverse	TGAAGGCGTTTGCACATGCA

### Tapestri GE pipeline performance metric definition

The performance of the pipeline v1.0 was assessed by comparing the editing status of each potential editing site (either on target or off target), as called by the pipeline, with the known truth for that site. Since the pipeline is capable of calling editing zygosity at each potential editing site, the editing status of each copy of the DNA (allele) was compared. The test cells were known to be diploid at each potential editing site, so this resulted in two possible events at each editing site (in each cell). We compared the known editing status for each target in each cell with the editing status called by the Tapestri pipeline. To quantify these comparisons, we used the following definitions of the different possible outcomes (Figure S11).

(1) TP: Allele is edited in known truth data, and the pipeline called as edited.
(2) TN: Allele is unedited in known truth data, and the pipeline called as unedited.
(3) FP: Allele is unedited in known truth data, and the pipeline called as edited.
(4) FN: Allele is edited in known truth data, and the pipeline called as unedited.

Since each cell is known to be diploid for each target (on target and off target) and the above definitions are for each allele, each target in each cell gives rise to two different events. Table 2 summarizes the two possible events for every combination of the known truth genotype and the Tapestri pipeline called genotype.

**Table 2:** Potential events for each combination of known truth genotype and GE called genotype

Expectation (truth)	Observation (pipeline call)	Events
WT	WT	TN, TN
WT	mono-allelic	TN, FP
WT	bi-allelic	FP, FP
Mono-allelic	WT	TN, FN
Mono-allelic	mono-allelic	TP, TN
Mono-allelic	bi-allelic	TP, FP
Bi-allelic	WT	FN, FN
Bi-allelic	mono-allelic	FN, TP
Bi-allelic	bi-allelic	TP, TP

These events were counted for each potential editing site in each cell for all the cells in a Tapestri run and used to calculate the sensitivity, specificity, FPR, FNR, and accuracy for that particular Tapestri run. The equations used to calculate these metrics are listed below.

(1) Sensitivity = TP/(TP + FN)
(2) Specificity = TN/(TN + FP)
(3) FPR = FP/(FP + TN)
(4) FNR = FN/(FN + TP)
(5) Accuracy = (TP + FN)/(TP + FN + FP + TN)

Each clonal sample was processed through Tapestri in triplicates (three Tapestri runs) and the performance metrics were calculated for each Tapestri run independently.

### Protein read normalization and analysis with DNA editing status

Protein read counts per cell were calculated by the Tapestri Protein pipeline. Once we had the distribution of protein reads across the 45 (or 47) antibodies for each single cell the read counts were normalized via a proprietary normalization method developed by Mission Bio. Any cells that had positive signals for control antibodies (immunoglobulin G1 [IgG1], IgG2a, and IgG2b) were removed from the analysis. Additionally, any cells that had an antibody signal for more than 38 out of 45 (or 47) antibodies were removed from the analysis. These cells are hypothesized to be sticky cells which produce FP (nonspecific) signals for antibodies. Once noisy protein cells were removed from the analysis, we clustered the cells using the Louvain method^55^ and annotated each cluster for its cell type using known antibody markers for PBMCs and the Jurkat cell line.

Cell types (Jurkat and donor PBMCs) were identified using germline variants in the targeted DNA panel. During this identification, cell barcodes which had mixed signals were also identified and removed. These are barcodes from droplets which had two encapsulated cells and hence have a mixed single nucleotide variant signature for germline variants from both cell lines.

The genotyping of gene-editing targets was performed by Tapestri GE pipeline, which produces an editing status per target per pipeline (as described in the Tapestri DNA + protein sequencing v3 user guide). After combining the gene-editing status information for the *TCRA* on-target site with the protein labels from protein analysis, we were able to detect the difference in CD3 expression for different editing zygosity on the *TCRA* on-target site.

### Single-cell data analysis and variant calling

FASTQ files generated by the Illumina Nextseq 550 sequencer are processed through the Tapestri GE Pipeline for adapter trimming, cell barcode extraction and correction, read alignment to genome, and cell calling. In this study, the data was were analyzed by the Tapestri GE Pipeline. Once cell calling is completed, the cells are run through the translocation identification and variant calling workflows.

The *TCRB* locus in the human reference genome (hg38) is repeated and the guide used can bind to two different locations which are separated by 9.3 kbp in the reference genome (chr7:142791992-142792033 and chr7:142801339-142801380) (Figure S12). Due to the high similarity in these regions, a single pair of primers was designed to amplify both on-target regions. This was deemed as the only feasible way to amplify both targets without creating unintended amplification products. During analysis, we observed a bias in the amplification performance of these target locations, where only the first site (chr7:142791992-142792033) had sufficient reads per cell (≥10 reads per cell) to call variants accurately. Due to the lack of amplification and sequencing reads on the second target (chr7:142801339-142801380) across cells, we were not able to accurately call variants for this target, and the target was classified as a no call and dropped from the analysis. Amplicon optimization is common in targeted resequencing-based technologies. Redesigning different iterations of primers to favor the second target is likely but was out of scope of this particular study.

Translocation identification is performed by checking for chimeric reads in each cell. Chimeric reads are defined as reads that begin with the primer of one amplicon and end with the primer of another. These amplicons must be from different chromosomes or at least 2 kb apart on the same chromosome (in case of intrachromosomal translocations). The pipeline uses two filters to minimize FPs when detecting translocation events.

(1) At least 10 reads must support a translocation event in a cell.
(2) The ratio of chimeric to normal reads must exceed 0.15 for a translocation event in a cell.

The chimeric to normal read ratio is calculated by dividing the number of chimeric reads (those spanning two amplicons) by the average number of properly paired reads for those amplicons in the same cell. This method assumes that when a translocation occurs in just one of the DNA copies in a cell, both chimeric and normal reads will be present. Since these reads come from the same DNA copy, their quantities should be similar. If only chimeric reads are found in a cell, this ratio-based filter is not applied since we assume that the translocation is a bi-allelic translocation affecting both copies of DNA in that cell.

Once translocation reads are separated from non-translocation reads or normal reads, these reads are used for variant calling, which is performed using GATK best practices workflow for each individual cell. Variants are first called using GATK HaplotypeCaller and then genotyped using the GenotypeGVCFs tool.^56^ This results in having both translocation and variant calling information for each potential editing site in each single cell.

To classify a potential editing site as edited or unedited in a single cell we first apply some preliminary filters on the variants called by GATK. These filters are as follows:

(1) Remove variants which have a GQ (as calculated by GATK) of less than 30.
(2) Remove variants which have a DP (as calculated by GATK) of less than 10.
(3) Remove variants which have an AF (allele frequency, as calculated by GATK) of less than 0.2.

Additionally, after applying these filters, we also remove any indels of length 1 bp that were present in only up to three cells per sample. These variants are assumed to be likely errors due to either sequencing or PCR. After variant filtering, we classify targets that have any indels as edited in a cell. We also utilize phasing information from GATK to phase together multiple variants (in the case that such variants are present) on the same target to categorize targets as mono-allelically edited vs. bi-allelically edited.

### Primary T cell samples

T cells were isolated from human PBMCs (purchased from Lonza) via a Human Pan T cell Isolation Kit (Miltenyi Biotec) according to the manufacturer’s instructions. Isolated T cells were subjected to activation by Dynabeads Human T-Activator CD3/CD28 (Gibco) according to manufacturer’s instructions, seeded at a density of 500,000 cells/mL, and cultured for 48 h in a complete growth media (RPMI-1640 + 10% FBS + penicillin/streptomycin [P/S]) supplemented with 30 U/mL interleukin 2 (IL-2). Afterward, the beads were removed on a magnetic rack, cells replated at a density of 500,000 cells/mL and kept in culture for an additional 24 h in a complete growth media (RPMI-1640 + 10% FBS + P/S) supplemented with 300 U/mL IL-2.

### Primary T cell editing

On the day of electroporation, gRNAs were generated by combining equivalent volumes of 200 μM crRNAs (TCRA/TCRB/PDCD1) and 200 μM tracrRNA. TracrRNA and crRNAs and were purchased from IDT (TCRA: UGUGCUAGACAUGAGGUCUA; TCRB: GGAGAAUGACGAGUGGACCC; PDCD1: GGCGCCCUGGCCAGUCGUCU). Annealing of crRNA and tracrRNA was performed at the thermocycler with the following program: 5 min 95°C + 14 cycles in increments of −5°C, 10 min 20°C, and the resulting gRNAs were kept on ice. RNPs of gRNA + Cas9 were prepared at a molar ratio of 1.2: 1: 120 pmol (1.2 μL) of each of three annealed gRNAs – 3.6 μL in a total +3 × 104 pmol (5.1 μL) of Alt-R S.p. Cas9 Nuclease V3 (IDT). The mixture was incubated for 15 min at 25°C to produce RNP. Meanwhile, the cells were harvested and centrifuged at 300×*g* for 5 min. After the supernatant was aspirated, the cell pellets were resuspended in 1 mL PBS, transferred to 1.7-mL tubes, and centrifuged again at 300×*g*. After removing supernatant, cell pellets were resuspended in 20 μL of P3 Primary Cell Solution (Lonza) + 1 μL of 100 μM Alt-R Cas9 Electroporation Enhancer (IDT) per 1 × 10^6^ cells, and 21 μL of cells were combined with 8.7 μL of RNP complex in the sterile 0.2 mL thin-wall PCR tubes (Axygen). The entire cell-RNP mixture was transferred into a well of 16-well nucleocuvette strip (Lonza) and electroporated at 4D-Nucleofector using the EO-115 program. Immediately after nucleofection, 75 μL of complete growth media were added to the electroporation wells, and cells were resuspended and transferred into a well of 24-well tissue culture dish with 2 mL of complete growth media supplemented with 300 U/mL IL-2. After the cells reached a total number of 5 × 10^6^ viable cells (5 days for donor 1 and 10 days for donor 2), they were harvested and the aliquots of 500,000 cells were collected, centrifuged for 2 min 1,000×*g* and the supernatant removed. Cell pellets were lysed in 100 μL of QuickExtract (Lucigen) according to the manufacturer’s protocol. The remaining cells were centrifuged at 300×*g* for 7 min, supernatant removed, cells resuspended in 1 mL of cold CryoStor CS solution (StemCell Technologies) per 1 × 10^6^ cells, and 1-mL aliquots were frozen in cryovials in LN2.

### PDCD1, TCRA, and TCRB off-target panel design

To identify potential off-target sites, GUIDE-seq experiments were conducted in a HEK293 cell line with stable Cas9 expression (HEK-Cas9) for each gRNA individually, as described previously^37^ (data not shown). Targeted amplification using rhAmpSeq was then performed on the top GUIDE-seq sites for each gRNA using the HEK-Cas9 system, as described previously.^37^ RhAmpSeq was also applied to triple-edited primary T cells (data not shown). Based on these experiments, a custom 115-plex panel was designed to encompass both on-target sites and top-ranking off-target sites for the Tapestri and rhAmpSeq assays. For Tapestri, amplicon design was performed using Tapestri Designer tool from Mission Bio, with amplicons ranging between 189 and 295 bp. For rhAmpSeq, amplicon design was performed using the IDT design tool (https://eu.idtdna.com/pages/tools/rhampseq-design-tool), with a size range of 160–350 bp. The primers and amplicons used to generate the Tapestri and rhAmpSeq panels are listed in Tables S3 and S4, respectively.

### Population-level on-target and off-target measurements

On-target and off-target CRISPR activity in the bulk cell population was determined by rhAmpSeq multiplex PCR as described previously.^37^ Sequencing was performed in a 2 × 150 bp paired-end format on the Illumina MiSeq platform, resulting in a total of 0.9–1.5 million reads per sample (see Table S2). Analysis of the indel percentage and translocation detection was performed via CRISPECTOR (v1.0.7).^38^ with default parameters. The configuration file used for the analysis can be found in Table S5.

## Data availability

scDNA-seq FASTQ files obtained from the cell line for the CRISPR editing vs. proteomic readout analysis, as well as H5 files, were deposited in NCBI under accession number PRJNA1154991. Raw sequencing data (bulk and single cell) obtained from the primary T cells are available in NCBI under accession number PRJNA1152481. H5 files generated through the Tapestri GE pipeline are accessible under the same BioProject. Any additional data are available from the corresponding authors upon reasonable request.

## Acknowledgments

The authors extend their gratitude to the members of the Hendel Lab and the Mission Bio team for their insightful feedback on the manuscript and for their invaluable contributions through enlightening discussions. Figures in the manuscript were created with BioRender.com. The authors declare that no financial support was received for the research, authorship, and/or publication of this article.

## Author contributions

C.L. and A.H. conceived and supervised the project. N.K., M.R., B.S., C.L., and A.H. contributed to the study design. M.R. prepared the samples and performed the bulk-sequencing experiments. Q.A., J.N., and C.L. performed the single cell experiments. S.G. and S.W. developed the computational GE pipeline. N.K. and S.G. analyzed the data. N.K., S.G., M.R., C.L., and A.H. wrote the manuscript with contributions from all authors. All authors read and approved the final manuscript.

## Declaration of interests

S.G., Q.A., J.N., S.W., B.S., and C.L. were employees of Mission Bio. A.H. is the founder and CSO of Cassidy Bio. However, Cassidy Bio does not have input into the design, execution, interpretation, or publication of the work in this manuscript.

## Declaration of generative AI in scientific writing

During the preparation of this work, the authors used available AI tools (e.g., Copilot and ChatGPT) to enhance the quality of our writing. After using these tools, the authors reviewed and edited the content as needed and take full responsibility for the content of the publication.

## Supplemental information

Document S1. Figures S1–S12 and Tables S1 and S2

Table S3. On-target and off-target Tapestri panel Genomic coordinates are provided relative to the hg38 reference genome.

Table S4. On-target and off-target rhAmpSeq panel Genomic coordinates are provided relative to the hg38 reference genome.

Table S5. CRISPETOR config file Config file used for running CRISPECTOR on the bulk-sequencing rhAmpSeq data.

Document S2. Tapestri DNA sequencing v3 user guide

Document S3. Tapestri DNA + protein sequencing v3 user guide

Document S4. Article plus supplemental information
