# Supplementary material for: Precise measurement of CRISPR genome editing outcomes through single-cell DNA sequencing
Source: Mol Ther Methods Clin Dev. 2025 Mar 14;33(2):101449. doi: 10.1016/j.omtm.2025.101449 (PMC11987616; doi:10.1016/j.omtm.2025.101449)
Supplement: Document S1. Figures S1–S12 and Tables S1 and S2 [file mmc1.pdf]

OMTM, Volume *33*

Supplemental information

Precise measurement of CRISPR genome editing outcomes through single-cell DNA sequencing

Nechama Kalter, Saurabh Gulati, Michael Rosenberg, Qawer Ayaz, Joanne Nguyen, Shu Wang, Benjamin Schroeder, Chieh-Yuan Li, and Ayal Hendel

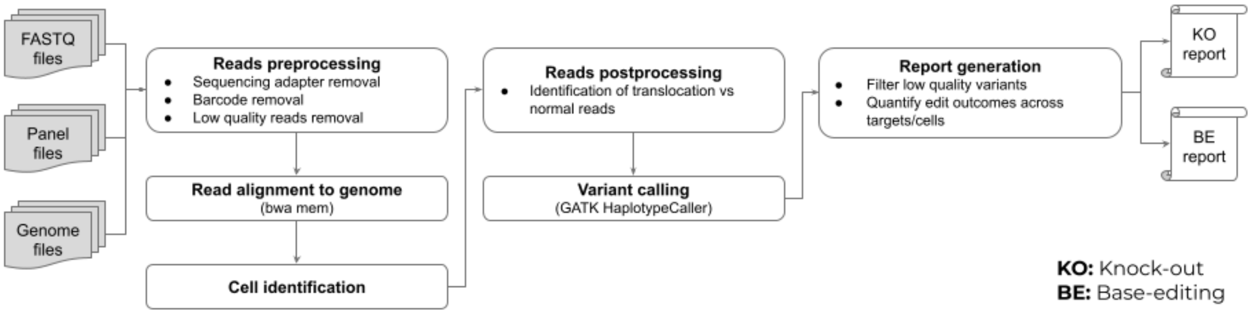

<u>Fig. S1. Flowchart and module description of the Tapestri GE pipeline</u>. The Tapestri GE pipeline accepts amplicon sequencing files, a reference genome sequence, and a panel file detailing the targets and amplicon coordinates. The pipeline then generates comprehensive reports tailored to the outcomes of KO and base-editing experiments.

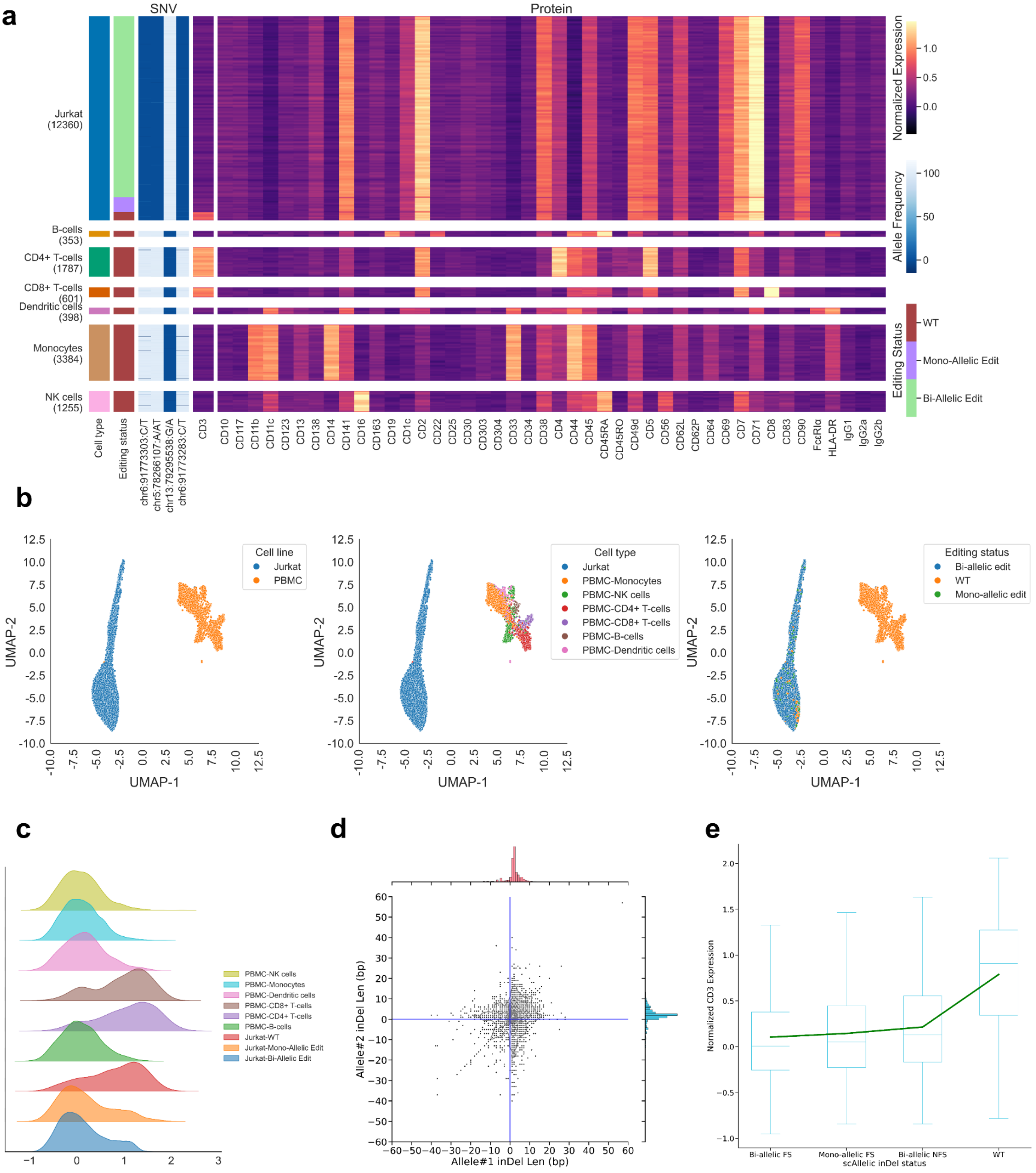

Fig. S2. Direct single-cell multi-omics measurement of editing genotype and cell surface proteins. **(a)** Heatmap depicting single-cell surface protein expression, editing status, and cell type classification derived using both sample-specific SNVs and protein markers. Specifically, samples in the heatmap include a mixed population (heterogeneous pool) of CRISPR–Cas9 *TCRA* edited Jurkat cells ($CD3^+$) and peripheral blood mononuclear cells (PBMCs) comprising various lineages. **(b)** UMAP

visualization of a cell in a Tapestri run, comprising a mixture of TRAC-edited Jurkat cells and unedited PBMCs. The Tapestri GE Protein + DNA pipeline provides per-cell SNV data, co-occurrence of edits, zygosity, and quantitative surface protein expression profiles across a total of 20,138 cells. The left UMAP displays distinct clusters corresponding to the Jurkat and PBMC samples, differentiated by sample-specific SNV signatures. The middle UMAP integrates immunophenotypic data, highlighting cell surface marker expression profiles from 45 key hematopoietic lineage markers. The right UMAP indicates the editing status of each cell, with unedited PBMCs labeled as WT and Jurkat cells categorized as WT, mono-allelically edited, or bi-allelically edited. **(c)** Ridge plot illustrating normalized CD3 expression across different cell types within PBMCs and Jurkat cells, stratified by TCRα editing status. **(d)** Single-cell, allele-specific indel length analysis at the *TCRA* locus. Each point on the plot represents a pair of inDel lengths on the two alleles, with the accompanying histogram and contour plot depicting the density distribution. Most edited cells exhibit balanced small inDels across both alleles, whereas a subset shows either imbalanced inDel lengths (e.g., allele 1: long, allele 2: short) or balanced large inDels on both alleles. **(e)** Box plot comparing surface CD3 expression levels with cell editing zygosity and knockout status, as determined by single-cell allele-specific indel length analysis. In line with previous studies, the data demonstrate that *TCRA* FS edits, both bi-allelic and mono-allelic, disrupt the CD3-TCR complex, leading to reduced CD3 surface expression. The green line connects the mean CD3 expression level within each editing group. FS, frameshift. NFS, non-frameshift.

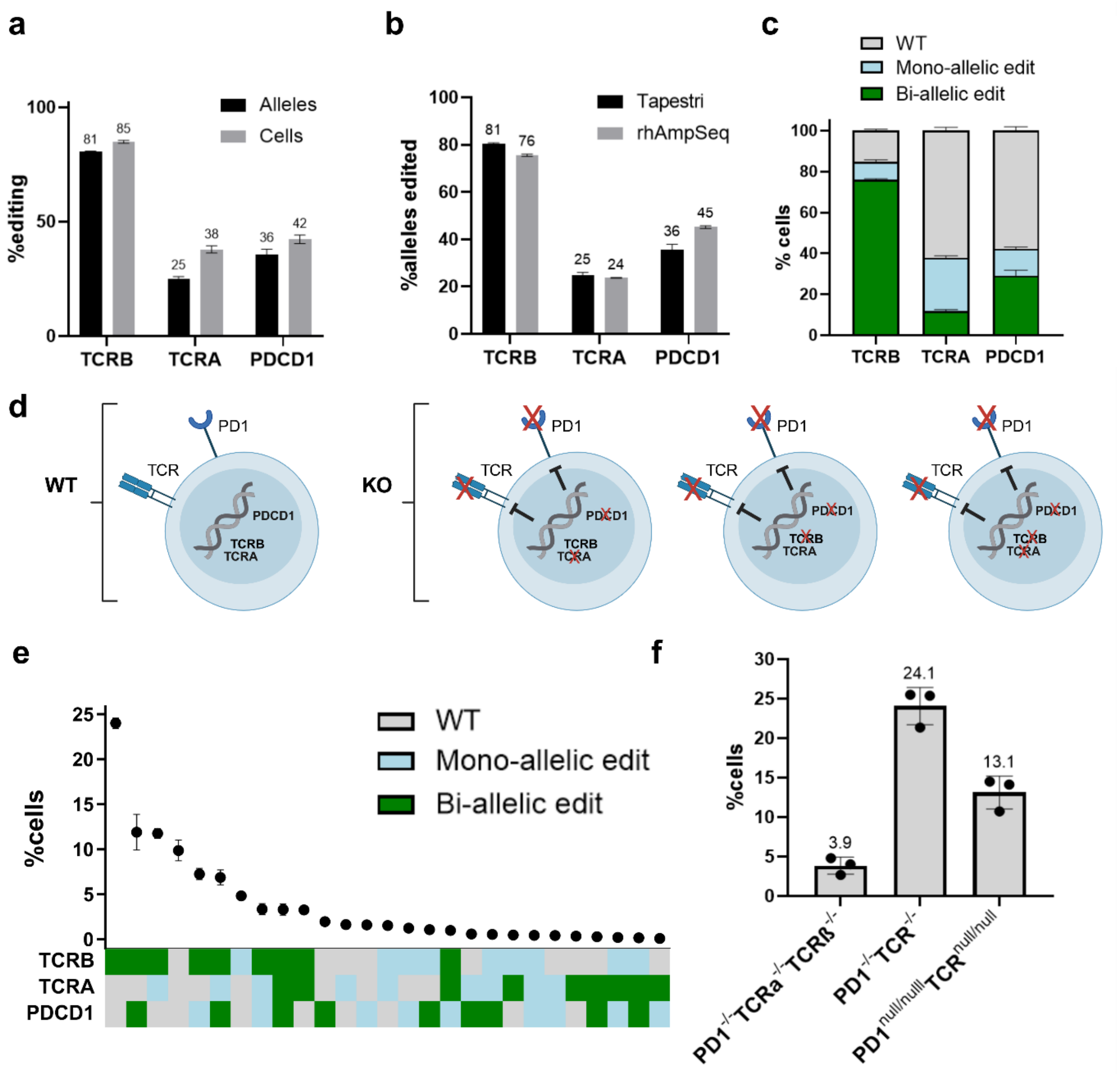

Fig. S3. Analyzing on-target CRISPR efficiency at single-cell resolution. Editing efficiency at the on-target sites of the second donor tested. **(a-b)** Editing activity at the on-target sites as determined by Tapestri (n = 3, 4-8.3K cells per sample) and rhAmpSeq (n = 2). **(a)** Tapestri results at each on-target site, at the allele and cell levels (black and gray bars, respectively). **(b)** Comparison between the Tapestri (black bars) and rhAmpSeq results (gray bars). **(c)** Zygosity of on-target edits, shown as the fraction of cells with a bi-allelic edit, mono-allelic edit, or no edit at the indicated locus (green, blue, and gray bars, respectively). **(d)** Representation of the target cell population, with a KO in the TCR and PD1 receptors. **(e)** Co-occurrence of editing events at on-target sites. **(f)** Fraction of the target cell population with a KO of TCR and PD1 receptors. Left bar, cells with a bi-allelic edit at all three target sites (PD1$^{-/-}$ TCRα$^{-/-}$TCRβ$^{-/-}$); middle bar, bi-allelic edit at the *PDCD1* locus and in at least one TCR gene (TCRα,

TCRβ, or both) (PD1$^{-/-}$TCR$^{-/-}$); right bar, FS bi-allelic edit at the *PDCD1* locus and in at least one TCR gene (PD1$^{null/null}$TCR$^{null/null}$). Error lines represent SD.

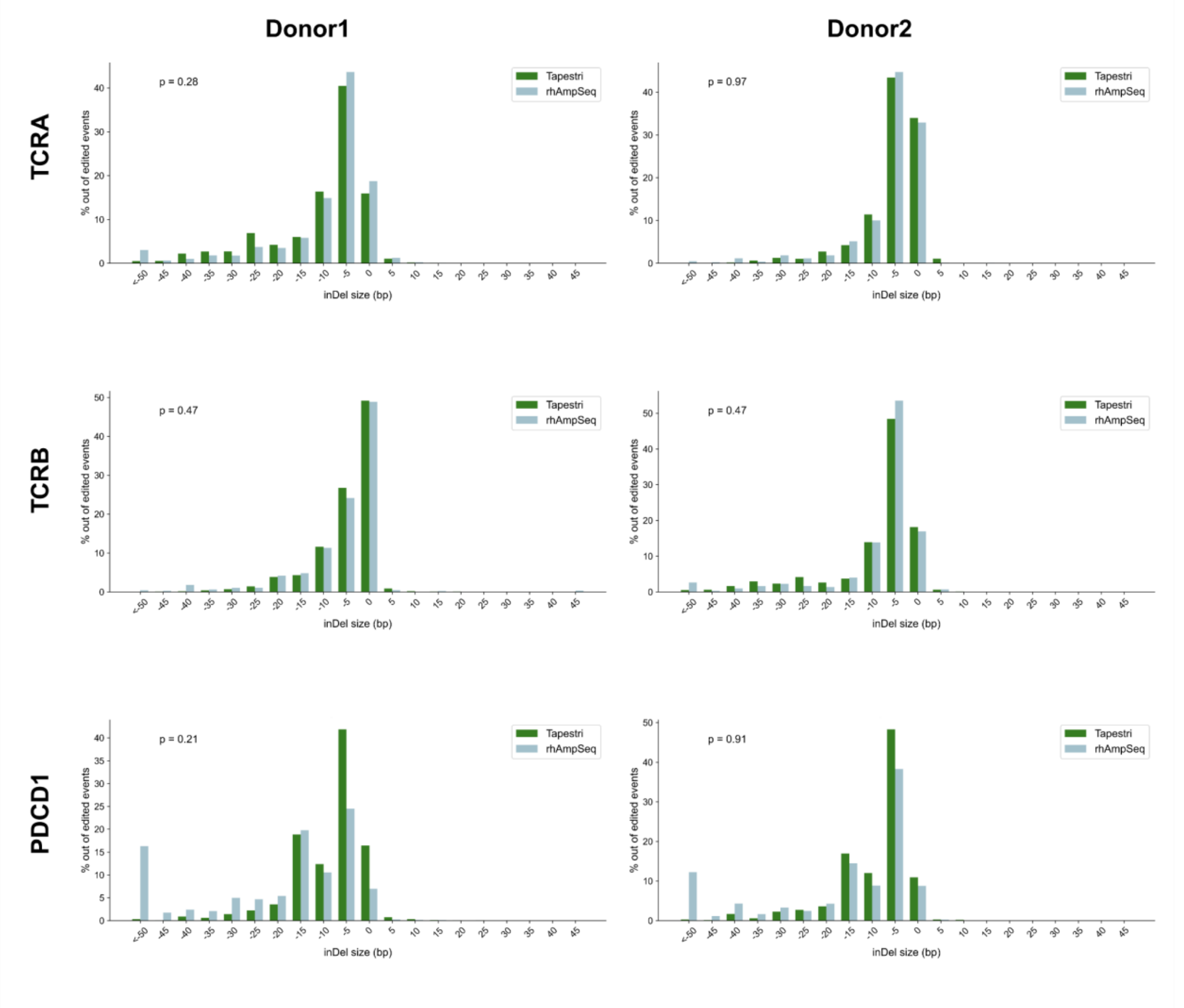

Fig. S4. Comparison of inDel size distribution between Tapestri and rhAmpSeq. InDel size distribution across on-target sites, as measured by Tapestri (green bars) and rhAmpSeq (blue bars), is shown for each of the on-target sites: *TCRA* (top row), *TCRB* (middle row), and *PDCD1* (bottom row). The left graphs represent donor 1, while the right graphs represent donor 2. The Kolmogorov–Smirnov test was applied to assess the similarity between the two assays for each target site, with the resulting p-value reported for each sample. The *PDCD1* on-target site exhibited an abundance of large deletions (>50 bp), particularly in sample D1.

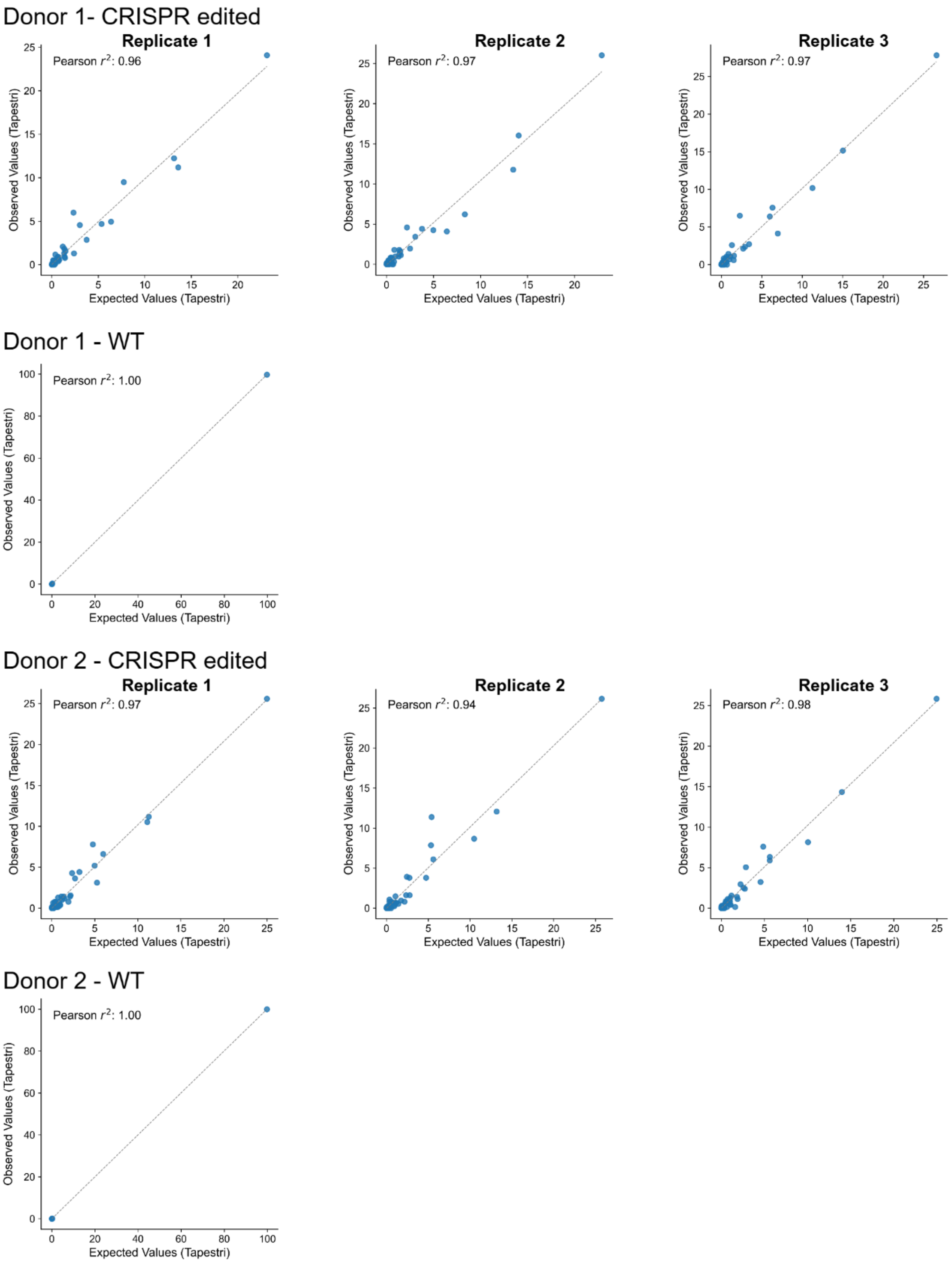

Fig. S5. Prediction of co-occurrence of editing combinations in multiplex edited cells based on target-specific results. Comparison between the expected and observed frequencies of co-occurrence combinations (WT, bi-allelic editing or mono-allelic editing) between the *TCRA*, *TCRB*, and *PDCD1* on-target sites, as well as the TCRB_OT-51 off-target site. Expected frequencies for each co-

occurrence combination were determined using pseudo-bulk allelic editing data derived from the single-cell experiments, as a probability of a cell having either WT, mono-allelic or bi-allelic editing status for a target. Simulations were performed for each target and matched to the number of cells analyzed in single-cell experiments (per sample). The simulated editing outcomes of multiple cells were then used to identify editing co-occurrence and compared to observed editing co-occurrence from sequencing data. Each plot compares co-occurrences of all tested sites (on-target sites and TCRB_OT-51) within a single sample. Pearson r^2 values and regression line (gray dotted line) are shown for each sample.

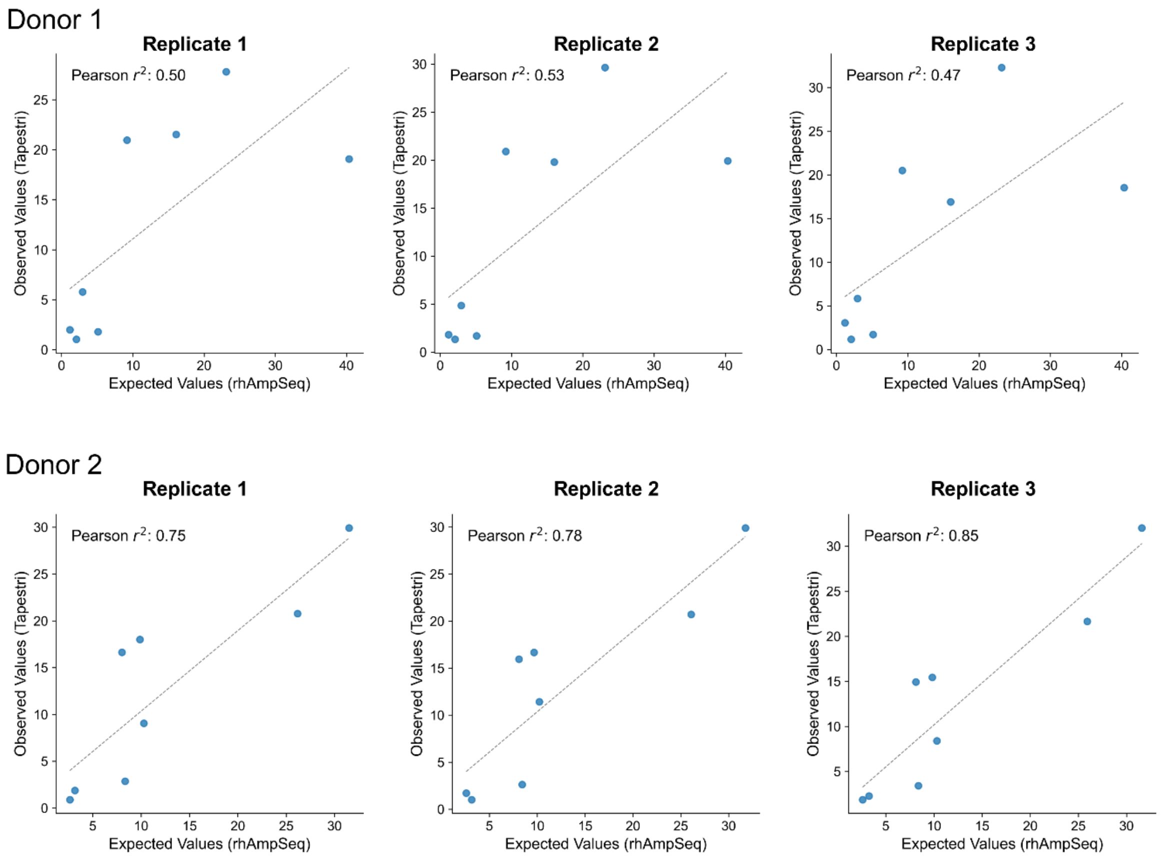

Fig. S6. Prediction of edited subpopulations using bulk-sequencing results. Comparison between the expected and observed frequencies of co-occurrence combinations (edited/unedited), across the *TCRA*, *TCRB*, and *PDCD1* on-target sites, based on the rhAmpSeq population-level analysis. To determine the expected values, we used the bulk sequencing editing frequency of each target (calculated by CRISPECTOR software), as the probability of an allele of a target being edited. We then simulated editing status for the three targets and the same number of alleles per sample as identified during single cell analysis (assuming all cells to be diploid for each target). The simulated editing statuses were used to identify different editing combinations and their frequencies (expected values) in the sample. These editing combinations were then compared to editing combinations identified from single-cell analysis (observed values). Pearson r^2 values and regression line (gray dotted line) are shown for each sample.

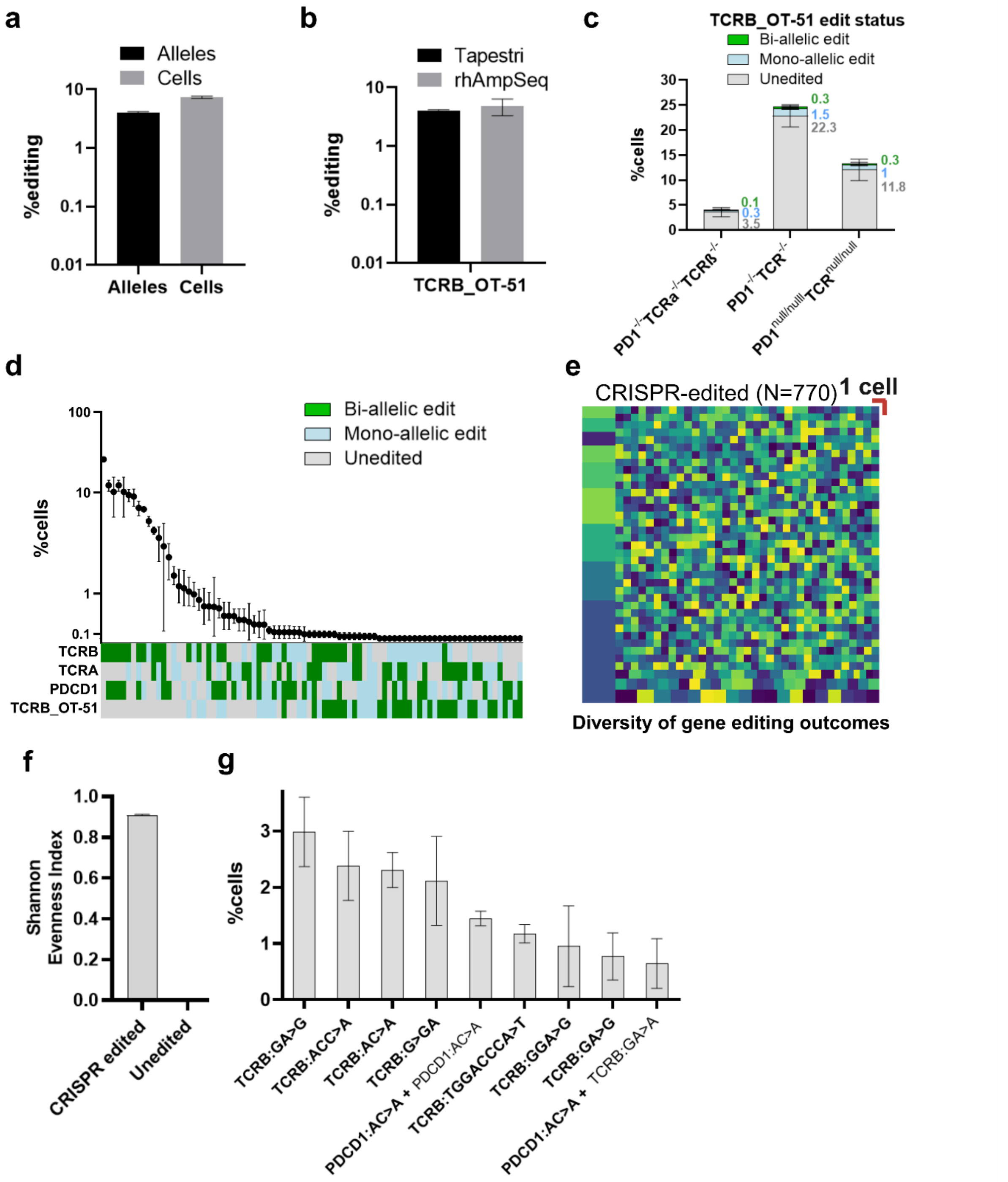

Fig. S7. Analyzing off-target CRISPR activity at single-cell resolution. Off-target activity measurement for the second donor tested. **(a-b)** Off-target activity as determined by Tapestri (n = 3, 4-8.3K cells per sample) and rhAmpSeq (n = 2). Sites with >0.1% editing in one of the methods are presented. **(a)** Tapestri-measured off-target editing activity at the allele and cell levels (black and gray bars, respectively). **(b)** Comparison between the Tapestri (black bars) and rhAmpSeq results (gray bars). N.A., Not detected by rhAmpSeq. **(c-d)** Co-occurrence of the most active off-target site (TCRB_OT-51) with desired on-target editing outcomes. **(c)** Fraction of cells with an off-target activity

in TCRB_OT-51 out of the target cells shown in **Fig. S3f.** **(d)** Co-occurrence of editing events at the on-target sites and TCRB_OT-51. **(e)** Treemap visualization showing the diverse editing outcomes, for a one replicate of edited cells. Each rectangle represents the fraction of cells with a specific combination of editing outcomes (different type of inDels) for each target, in each allele. The size of each rectangle corresponds to the relative abundance of that cell population. For reference, one of the smallest rectangles, representing a single cell, is marked with a red scale in the figure, while larger rectangles indicate proportionally higher cell numbers. **(f)** Shannon Evenness Index was used to assess the diversity of editing outcomes in the samples. The left bar represents the average Shannon Evenness Index for CRISPR-edited samples, while the right bar shows the value for the WT sample. The treated samples displayed a high diversity of editing outcomes, as indicated by an elevated average Shannon Evenness Index of 0.9(0.005). **(g)** Most frequent 'editing outcomes' observed in the edited population, out of the clones shown in Fig. S7e. with the specific inDels observed for that clone detailed. Bi-allelic inDels are indicated in bold font, and mono-allelic edits are shown in Roman font. The data are presented as mean (SD).

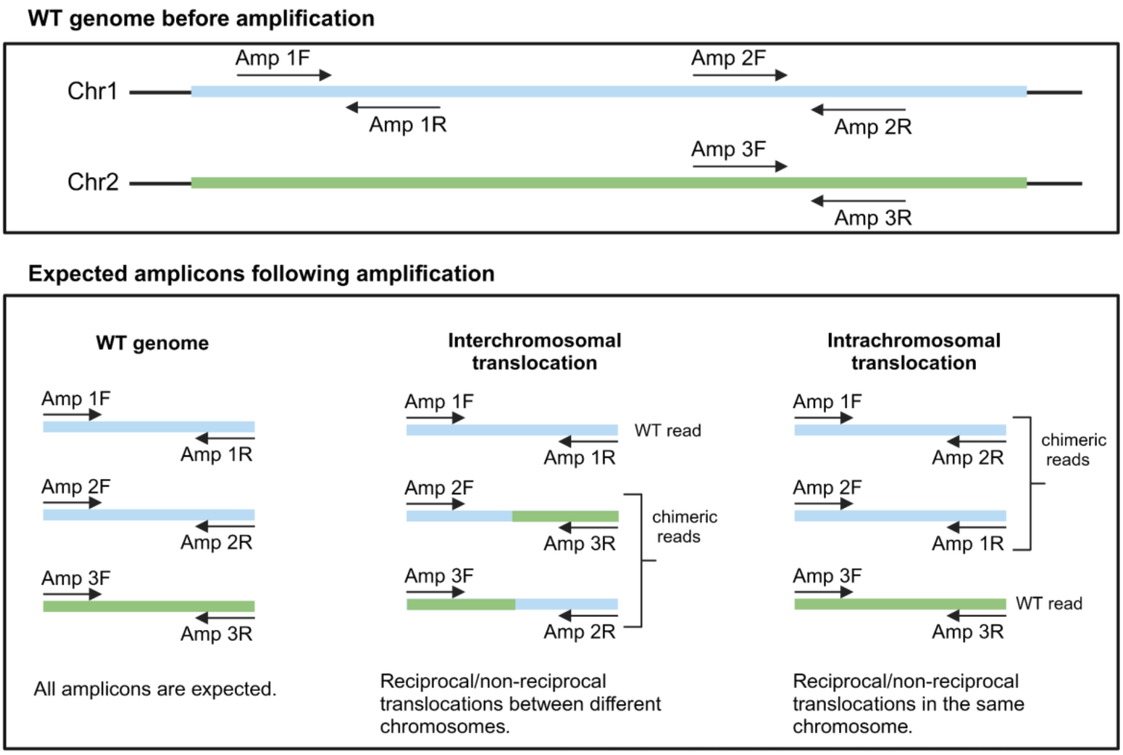

Figure S8. Translocation detection by the Tapestri technology. Illustration showing the possible translocation products detected by Tapestri. Translocation identification involves detecting chimeric reads, with primers from different amplicons at each end of each cell. To assess translocations, the ratio of chimeric to normal reads is calculated by dividing the number of chimeric reads by the average number of properly paired reads for the corresponding amplicons in the same cell (see Materials and Methods for more information). Tapestri can identify interchromosomal translocations between two different chromosomes (shown in blue and green in this illustration), or intrachromosomal translocations (within the same chromosome). Amp, amplicon. WT, wild-type. F, forward primer. R, reverse primer.

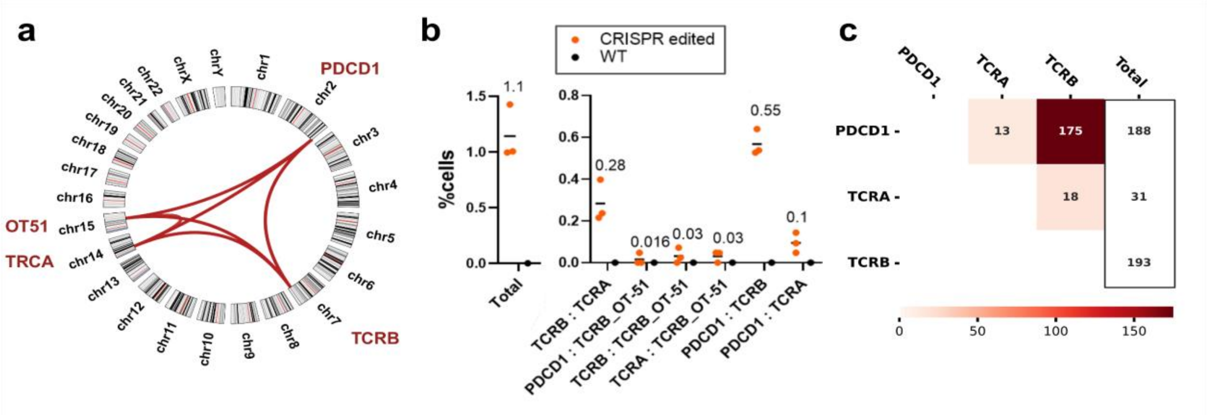

Fig. S9. Using single-cell sequencing for translocation detection in editing experiments. SV analysis of the second donor tested. **(a)** Circos plot showing translocations identified within the multiplex-edited population using single-cell sequencing. **(b)** Overall percentage of cells harboring translocations, as identified through single-cell sequencing (left), and distribution of specific translocation events within the cell population (right) (n = 3, 4-8.3K cells per sample). Mean values are presented. **(c)** Translocations validated by the bulk NGS. Numbers indicate read count for each translocation event in both replicates combined.

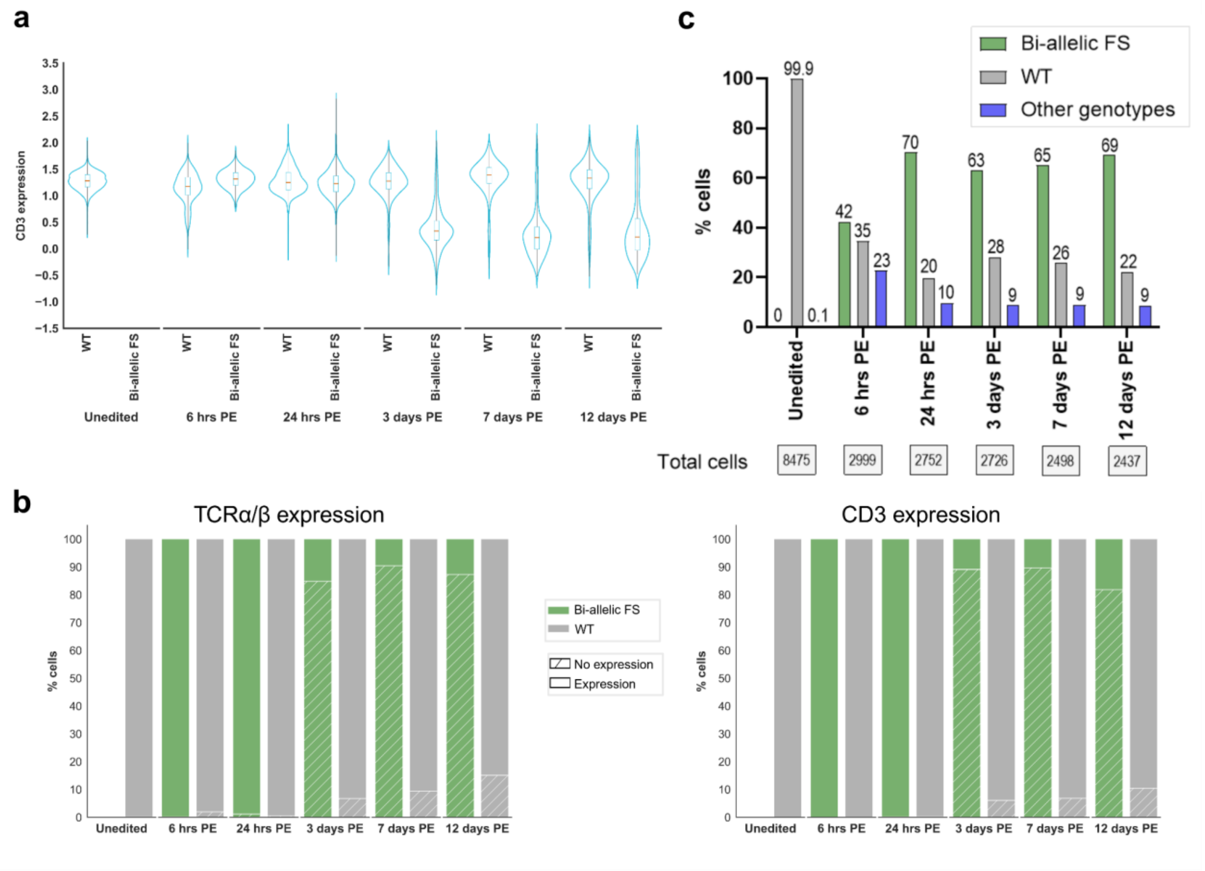

<u>Fig. S10. scDNAseq and proteomic analysis validate functional KO in edited primary cells.</u> (a) Violin plots depicting the CD3 cell surface expression for the timepoints tested. For each timepoint, cells are categorized using the following definitions: WT (cells with no *TCRA* or *TCRB* editing), and Bi-allelic frameshift (cells harboring a bi-allelic frameshift edit on either *TCRA* or *TCRB*). **(b)** Percentage of cells with no TCRα/β (left) or CD3 expression (right), at each timepoint. **(c)** Number of cells for each subpopulation of genotypes. Green bar, bi-allelic FS mutation; Gray bar, WT cells; Blue bar, mixed population with other genotypes. PE, post-editing. FS, frameshift.

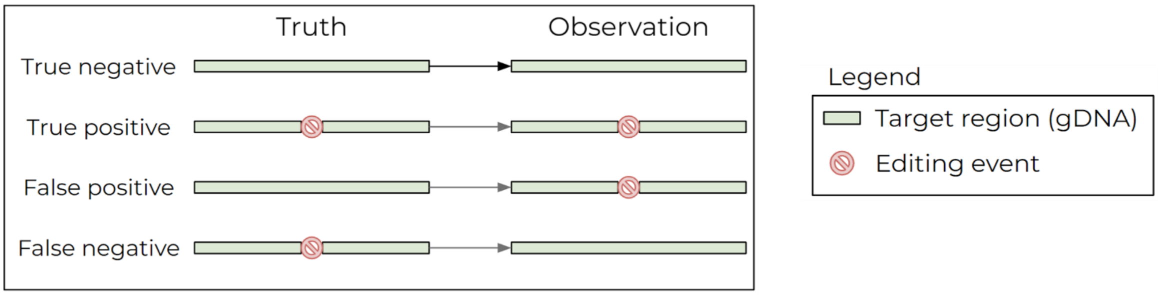

<u>Fig. S11. Performance assessment of the GE pipeline.</u> Demonstration of possible events (TN, TP, FP and FN).

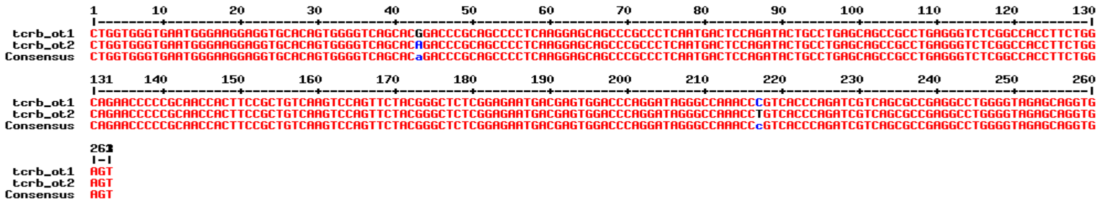

<u>Fig. S12. Sequence similarity between two TCRB on-target sites.</u> Sequences of the two on-target sites targeted by the TCRB gRNA are provided. The second on-target, here termed TCRB_OT-2, was excluded from this analysis to maintain high accuracy.

Table S1. Tapestri total sequencing reads per each T-cell sample. Total number of cells and sequencing reads per each primary T-cell sample sequenced by the Tapestri platform. Three technical replicates were performed per each treated sample, as well as an untreated control.

Sample	Total cells	Total read pairs	Read pairs assigned to cells	Read pairs assigned to cells (%)
Donor 1 treated - replicate 1	10603	239,069,248	148,118,728	61.96
Donor 1 treated - replicate 2	7366	173,446,994	100,654,406	58.03
Donor 1 treated - replicate 3	14105	206,727,387	125,449,112	60.68
Donor 1 untreated	10341	136,031,701	78,898,241	57.99
Donor 2 treated - replicate 1	4276	235,592,645	130,217,342	55.27
Donor 2 treated - replicate 2	4176	169,239,340	102,379,751	60.49
Donor 2 treated- replicate 3	4220	194,957,539	119,575,606	61.33
Donor 2 untreated	8376	179,954,635	104,417,180	58.02

Table S2. rhAmpSeq total sequencing reads per each T-cell sample. Number of sequencing-reads per each primary T-cell sample sequenced using the rhAmpSeq assay. Two technical replicates are shown per treatment or WT sample.

Sample	Total read pairs	Read pairs assigned to cells	Read pairs assigned to cells (%)
Donor 1 replicate 1 – treatment	947,492	906,180	95.64
Donor 1 replicate 1 – WT	815,653	783,815	96.10
Donor 1 replicate 2 – treatment	980,359	890,424	90.83
Donor 1 replicate 2 – WT	1,012,416	955,128	94.34
Donor 2 replicate 1 – treatment	1,646,257	1,567,295	95.20
Donor 2 replicate 1 – WT	1,326,684	1,268,276	95.60
Donor 2 replicate 2 – treatment	1,718,190	1,544,836	89.91
Donor 2 replicate 2 – WT	1,653,539	1,548,627	93.66

Table S3. On-target and off-target Tapestri panel. Genomic coordinates are provided relative to the hg38 reference genome.

Table S4. On-target and off-target rhAmpSeq panel. Genomic coordinates are provided relative to the hg38 reference genome.

Table S5. CRISPETOR config file. Config file used for running CRISPECTOR on the bulk-sequencing rhAmpSeq data.

Tapestri DNA Sequencing v3 User Guide.

Tapestri DNA + Protein Sequencing v3 User Guide.
