# Supplementary material for: Precise measurement of CRISPR genome editing outcomes through single-cell DNA sequencing
Source: Mol Ther Methods Clin Dev. 2025 Mar 14;33(2):101449. doi: 10.1016/j.omtm.2025.101449 (PMC11987616; doi:10.1016/j.omtm.2025.101449)
Supplement: Document S2. Tapestri DNA sequencing v3 user guide [file mmc5.pdf]

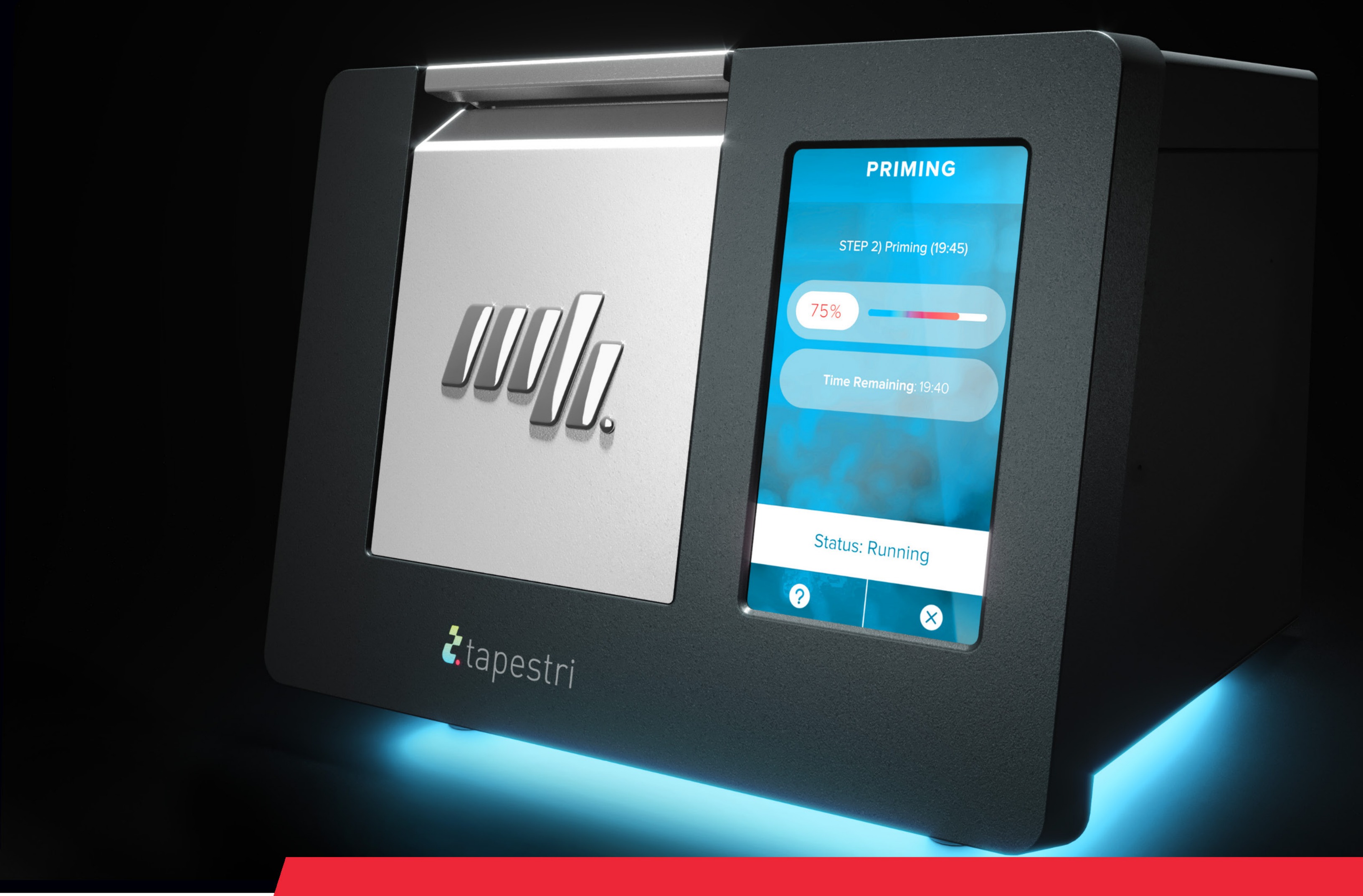

tapestri

Tapestri® Single-Cell DNA Sequencing v3

User Guide

mission bio

MISSIONBIO.COM

For Research Use Only. Not for use in diagnostic procedure.

Information in this publication is subject to change without notice. It is Mission Bio policy to improve products as new techniques and components become available. Therefore, Mission Bio reserves the right to change specifications at any time. Every effort has been made to avoid errors in the text, diagrams, illustrations, figures, and screen captures. However, Mission Bio assumes no responsibility for any errors or omissions. In no event shall Mission Bio be liable for any damages in connection with or arising from the use of this publication.

Trademarks.

© 2023 Mission Bio, Inc. All rights reserved. Duplication, public display, public performance, modification, or any other use of all or any portion of this document without the express prior written consent of Mission Bio is strictly forbidden. "Mission Bio" and the "MB" logo are trademarks of Mission Bio and may not be used for any purpose without the express prior written consent of Mission Bio. All other trademarks are the property of their respective owners.

Nothing in this documentation shall constitute a warranty, express or implied, as to the products (whether a product of Mission Bio, its affiliates, or any third party) or any protocols described herein, whether as to performance, accuracy, sufficiency, non-infringement, or otherwise. All warranties, if any, are set forth in the applicable terms and conditions of sale accompanying the purchase of Mission Bio's product. Without limiting the forgoing, Mission Bio and its affiliates provide no warranty and hereby disclaim any and all warranties as to the use of any third party products or protocols described herein.

The use or resale of the products described herein may be subject to certain restrictions as set forth in the applicable terms and conditions of sale accompanying the purchase of such product.

All products and services described herein are intended FOR RESEARCH USE ONLY and NOT FOR USE IN DIAGNOSTIC PROCEDURES.

Contact.

Mission Bio, Inc.
400 East Jamie Ct, Suite 100
South San Francisco, CA 94080
USA
www.missionbio.com

Table of Contents

Introduction 3
Tapestri Platform Overview 3
Materials. 5
Protocol Overview 8
Best Practices: Emulsion & DNA Cartridge 9
Thermal Cycling Programs 11
Cell Handling Guidelines 12
Genomic Protocol 13
1. Prepare Cell Suspension 14
2. Encapsulate Cells 16
3. Lyse and Digest Cells 20
4. Barcode Cells 22
5. Targeted PCR Amplification 27
6. Cleanup PCR Products 30
7. Library PCR 34
8. Quantify and Normalize Sequencing Library 38
9. Sequence Library 41
Sequence Information for Library Indices 1 – 8 43
Cartridge Map 43
Kit Contents 44
Tapestri Instrument Specifications 45
References 46

Introduction

The *Mission Bio Tapestri® Platform* uses microfluidic droplet technology to combine single-cell lysates with barcoding beads and gene specific primers to deliver a high-throughput single-cell genomics workflow for targeted DNA sequencing. Users can produce a sequencing-ready library starting from a single-cell suspension in as few as 2 days. This User Guide describes the experimental procedure in detail.

Tapestri® Platform Overview

The *Mission Bio Tapestri® Platform* consists of the instrument itself, the DNA cartridge, which represents the microfluidics device, and the reagents. The cartridge is equipped with reservoirs that are used to load reagents required for automated cell processing. Pressure supplied by the instrument drives the reagents from the reservoirs through the microfluidic device and out to PCR collection tubes that are mounted below the cartridge. The cartridge and tubes can be loaded and unloaded from the instrument and disposed of after the completion of the workflow. The user interacts with the instrument via a touch screen interface, which can be used to select programs, monitor the status of running programs, and more.

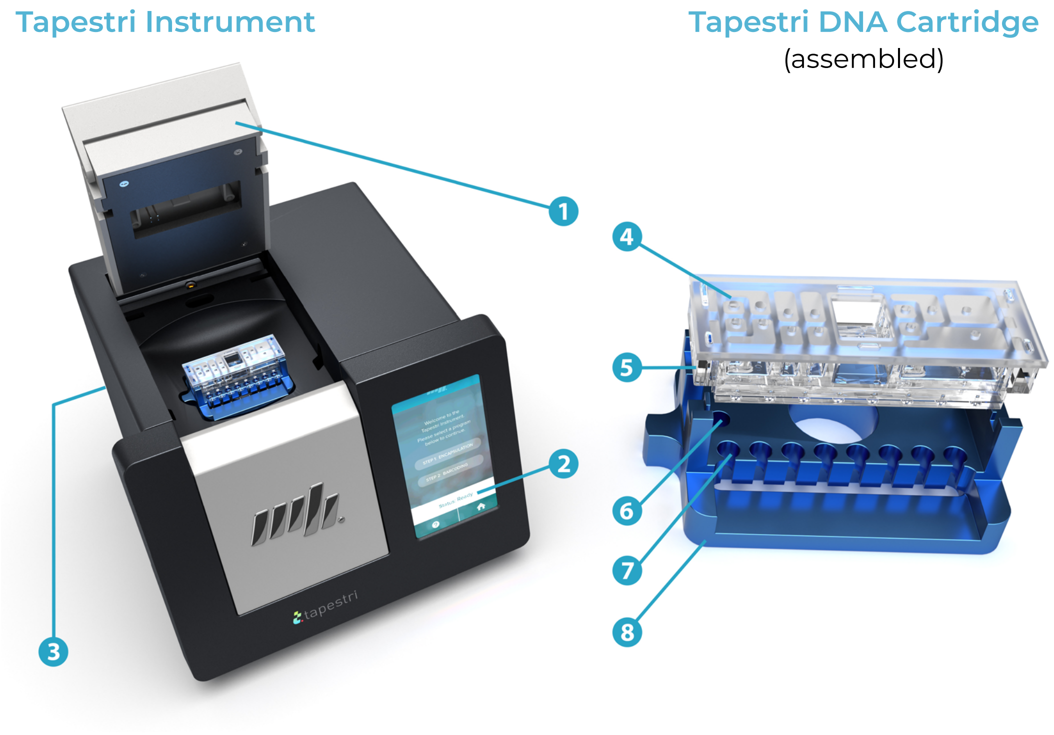

Figure 1. Tapestri Platform: Instrument and Assembled DNA Cartridge.

1. **Lid**
   Levered lid to open and close the instrument and install the DNA Cartridge.

2. **Touchscreen**
   To interface with the instrument's software and select programs.

3. **USB Port**
   To export diagnostics data.

4. **Tapestri DNA Gasket**
   To seal the catridge.

5. **Tapestri DNA Cartridge**
   Microfluidics device to load with reagents and cells.

6. **Encapsulation Collection Tube Slot**
   To collect encapsulation emulsions.

7. **Barcoding Collection Tube Slots**
   To collect barcoding emulsions.

8. **Base Plate**
   Foundation to mount DNA Cartridge and collection tubes.

Materials

Tapestri Single-Cell DNA Core Kit v3 Configuration

Component Name	Part Number	Storage
Tapestri Single-Cell DNA Core +4 Kit v3	MB03-0092	4°C
Tapestri Single-Cell DNA Core -20 Kit v3	MB03-0091	-20°C
Tapestri Single-Cell DNA Bead Kit v3	MB03-0093	-20°C

Tapestri Single-Cell DNA Core Kit v3 Components

Component Name	Kit	Storage
Cell Buffer	Tapestri Single-Cell DNA Core +4 Kit v3	4°C
Encapsulation Oil		4°C
Electrode Solution		4°C
Barcoding Oil		4°C
● Extraction Agent (green cap)		4°C
● Lysis Buffer (brown cap)	Tapestri Single-Cell DNA Core -20 Kit v3	-20°C
Barcoding Mix		-20°C
● Library Mix (green cap)		-20°C
● DNA Clean up Buffer (white cap)		-20°C
● Clean up Enzyme (pink cap)		-20°C
● Library Indices 1 – 8 (purple cap)		-20°C
● Barcoding Beads (blue cap)	Tapestri Single-Cell DNA Bead Kit v3	-20°C

NOTE ***Make sure to use non-frost free freezers for all -20°C reagent storage.***

Required Third Party Consumables and Reagents

Component Name	Suggested Supplier (Part Number)	Protocol Step
AMPure XP Reagent	Beckman Coulter (A63880)	Targeted PCR, Library PCR
DPBS w/o $Ca^{2}+/Mg^{2}+$ (1X)	Gibco (14190-144)	Encapsulation
Qubit® dsDNA HS Assay Kit	Qubit® (Q32851)	Targeted PCR
Ethanol, Molecular Biology Grade	Sigma (E7023) or Approved Supplier	AMPure purification
Agilent DNA 1000 Kit or Agilent DNA High Sensitivity Kit	Agilent Technologies (5067-1504) Agilent Technologies (5067-4626)	Post-PCR quantitation
Trypan Blue	Thermo Fisher (15250061) or Approved Supplier	Dead cell staining
TipOne RPT ultra low retention filter tip	USA Scientific (1180-8810) or Approved Supplier	Liquid handling
Nuclease free Microcentrifuge Tubes, 1.5 mL	Eppendorf (0030108418) or Approved Supplier	Cell/Reagent handling
* 0.2 mL Emulsion safe PCR tubes	USA Scientific (1402-4700) or Axygen (PCR-02-L-C) or Axygen (PCR-02D-L-C)	Emulsion handling
200 uL Gel Loading Pipette Tips	Axygen (TGL200RD57R) or Approved Supplier	Emulsion handling
0.2 mL PCR Tubes	USA Scientific (1402-4708) or Approved Supplier	Non-emulsion PCR
Qubit Assay Tubes	Thermo Fisher (Q32856)	Post-PCR quantitation
Fetal Bovine Serum, certified, heat inactivated, United States	Thermo Fisher (10082139) or Approved Supplier	AMPure purification
15 mL LoBind conical tubes	Eppendorf (30122208) or Approved Supplier	Sample Preparation
KAPA Library Quantification Kit Illumina Platforms (OPTIONAL)	KAPA (KK4873)	Sequencing
Sequencing Reagent Kit 300 cycles (150bp PE) (MiSeq, HiSeq 2500, HiSeq 4000, NextSeq 550, NovaSeq 6000, NovaSeq X, NextSeq 2000)	Illumina	Sequencing

NOTE **** These consumables are used for handling emulsion samples and must not be substituted. Only listed consumables have been validated by Mission Bio.***

Required Benchtop Equipment

Required Equipment	Suggested Supplier (Part Number)
MB Tapestri® Instrument	Mission Bio (191335)
Countess® II Automated Cell Counter or equivalent	Thermo Fisher (AMQAX1000)
Agilent 2100 Bioanalyzer or Tapestation	Agilent (G2939BA), (G2992AA), (G2991BA)
Qubit Fluorometer	Qubit: Thermo Fisher (Q33216)
Pipettes, 1 µL – 1000 µL	Mettler-Toledo, Rainin Pipettes, or Approved Supplier
Microcentrifuge (1.5 mL, 0.2 mL PCR tubes)	Thermo Fisher (75004081) or Approved Supplier
Tube Vortexer	Thermo Fisher (88880017TS) or Approved Supplier
Thermal cycler with heated lid (100 µL volume, needs to support ramp rates between 1°C/s – 4°C/s)	Thermo Fisher (A24811) or Approved Supplier
0.2 mL 8-strip PCR tube Magnetic Separation Stand	Seqmatic (TM-700) or Approved Supplier
1.5 mL tube Magnetic Separation Rack	New England Biolabs (S1506S) or Approved Supplier

Protocol Overview

Single cells are individually partitioned into nanoliter droplets. Barcoding Beads and PCR reagents are introduced using the Mission Bio Tapestri Instrument and DNA Cartridge. Cell lysis, protease digestion, cell barcoding and targeted amplification using multiplexed PCR occur within the droplets. Droplets are then disrupted, and barcoded DNA is extracted for library amplification. Final libraries are purified and can be sequenced on one of the supported Illumina Sequencer instruments.

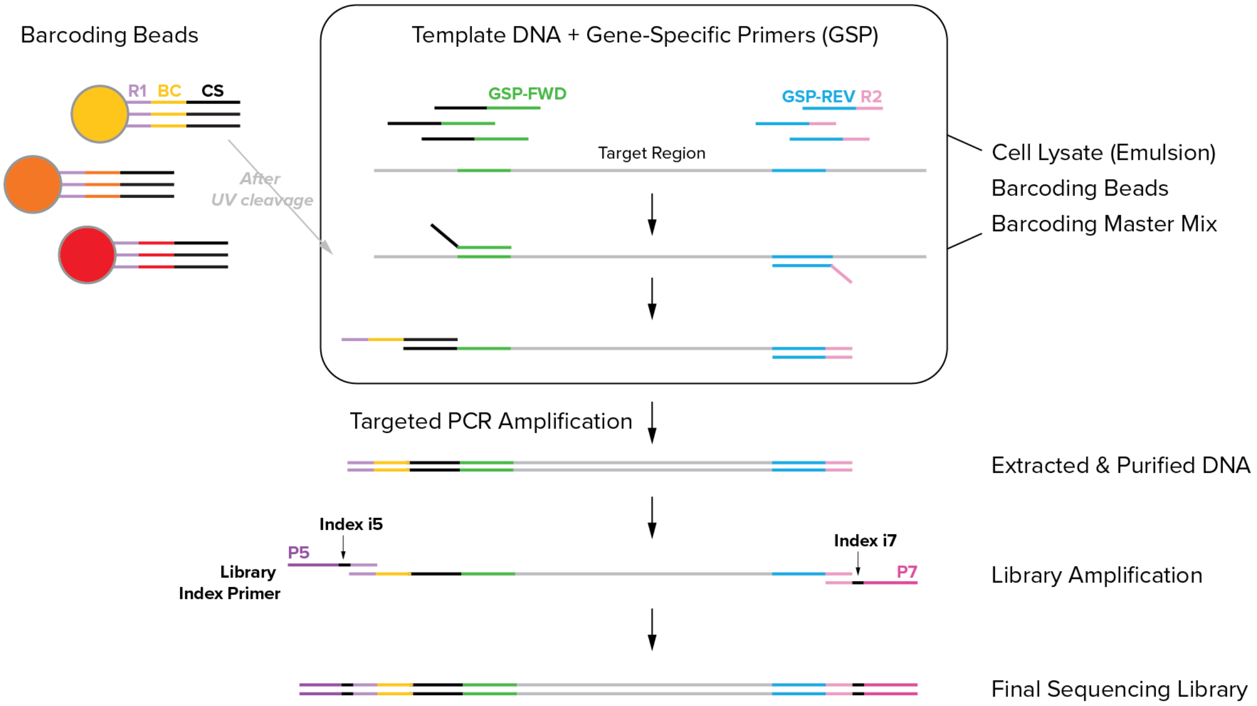

Figure 2. Overview of library construction. R1: Read 1, BC: barcode, CS: common sequence, GSP-FWD: gene-specific forward primer, GSP-REV: gene-specific reverse primer, R2: Read 2, P5: P5 Illumina adapter, P7: P7 Illumina adapter.

Best Practices: Emulsion & DNA Cartridge

Cell Culture, Pre- and Post-PCR areas

- All cell sample preparation must be conducted in a designated area that is restricted to cell culture work.
- All Pre-PCR steps (encapsulation, PCR master mix preparation, barcoding) must be conducted in a lab space that is physically separated from amplified genetic material.
- All Post-PCR (amplified material) steps (targeted PCR, library PCR, library purification, DNA quantification, sample pooling) must be conducted in a lab space that is physically separated from the unamplified genetic material.
- Do not transfer material (gloves, pipettes, tubes) or equipment from the Post-PCR area to the Pre-PCR area.
- Carefully clean bench areas and pipettes with 5% bleach before starting any protocol.

Cross-contamination

- When pipetting samples, change tips between samples.
- Use aerosol-resistant (filtered) pipette tips to reduce the risk of reagent carryover and sample-to-sample cross-contamination.

Suggestions for working with emulsions

- Consumables (gel tips, emulsion safe PCR tubes) have been carefully tested and specified. Do not substitute.
- Pipette emulsions very slowly and carefully and only when necessary.
- Avoid sources of static and any excess handling of emulsion samples.
- Handle emulsion sample tubes carefully. Avoiding direct contact with the sidewall of the tube, where emulsions directly interface, and hold tubes on the lid instead.

Suggestions for working with the Tapestri Instrument and DNA Cartridge

- Avoid introduction of particles, fibers or clumped cells into the cartridge that may potentially clog the cartridge.
- Minimize exposure of the instrument, reagents, cartridges, and gaskets to sources of particles and fibers, such as open reagent reservoirs, laboratory wipes, clothing that easily sheds fibers, and dusty surfaces.

- Place DNA cartridges into their original packaging after Encapsulation or Barcoding is completed.
- Lower the instrument lid when DNA cartridges are mounted on the instrument and are not in use.
- Pay attention to the timing of loading the DNA cartridge and running the Encapsulation or Barcoding programs. Experimental steps should be executed successively as outlined in the protocol without delays.
- Ensure that the instrument is not placed near a ventilation system or similar sources of high airflow.
- For additional information about requirements of the instrument's placement consult the *Tapestri Instrument Site Requirements Guide (PN 65307).*

Thermal Cycling Programs

Always use a properly calibrated thermal cycler suited for 0.2 mL tubes with a minimum reaction volume of 100 µL for all incubations. For all protocols, use a heated lid set to 105 °C and always use an adapter tray. For specific instrument operation, follow the instructions provided by the manufacturer.

1. Cell Lysis and Protein Digest		
**Step**	**Temperature**	**Time**
1	50 °C	60 min
2	80 °C	10 min
3	4 °C	HOLD

			2. Targeted PCR				
		Amplicon Number	**20 – 100**	**100 – 200**	**200 – 300**	**> 300**	
**Step**	**Ramp Rate**	**Temperature**	**Time**	**Time**	**Time**	**Time**	**Cycle**
1	4 °C/s	98 °C	6 min	6 min	6 min	6 min	
2	1 °C/s	95 °C	30 sec	30 sec	30 sec	30 sec	10
3		72 °C	10 sec	10 sec	10 sec	10 sec	
4		**61 °C**	3 min	4.5 min	6 min	9 min	
5		72 °C	20 sec	20 sec	20 sec	20 sec	
6	1 °C/s	95 °C	30 sec	30 sec	30 sec	30 sec	10
7		72 °C	10 sec	10 sec	10 sec	10 sec	
8		**48 °C**	3 min	4.5 min	6 min	9 min	
9		72 °C	20 sec	20 sec	20 sec	20 sec	
10	4 °C/s	72 °C	2 min	2 min	2 min	2 min	
11		4 °C	HOLD	HOLD	HOLD	HOLD	

3. Library PCR			
**Step**	**Temperature**	**Time**	**Cycle**
1	95 °C	3 min	
2	98 °C	20 sec	10
3	62 °C	20 sec	
4	72 °C	45 sec	
5	72 °C	2 min	
6	4 °C	HOLD	

Table 1. Thermal cycling programs.

Cell Handling Guidelines

The steps provided in this protocol are applicable to non-adherent cells from culture, bone marrow aspirates and buffy coat fractions. If other cell types will be used, contact ***support@missionbio.com*** for additional support. Different cell types may require revised procedures including cell dissociation, washing, re-suspension or quantitation.

Cell counting

- Mission Bio strongly recommends the use of an automated cell counter, such as the Countess II Automated Cell Counter (Thermo Fisher).
- Optimal concentration range for cell counting with the Countess II ranges from 1 x 10^5 to 4 x 10^6 cells/mL.
- Final cell suspensions are measured using at least two fields of view. Concentrations found must agree within 10%.
- Cell suspensions must have > 80% viability.
- Final cell concentration values are based on the **total (live + dead)** cell counts.
- Avoid the use of samples containing significant debris, dead cells, or fragments of lysed cells.
- Example images of a well-prepared single cell suspension (left) and low-viability cell suspension (right) are shown below.

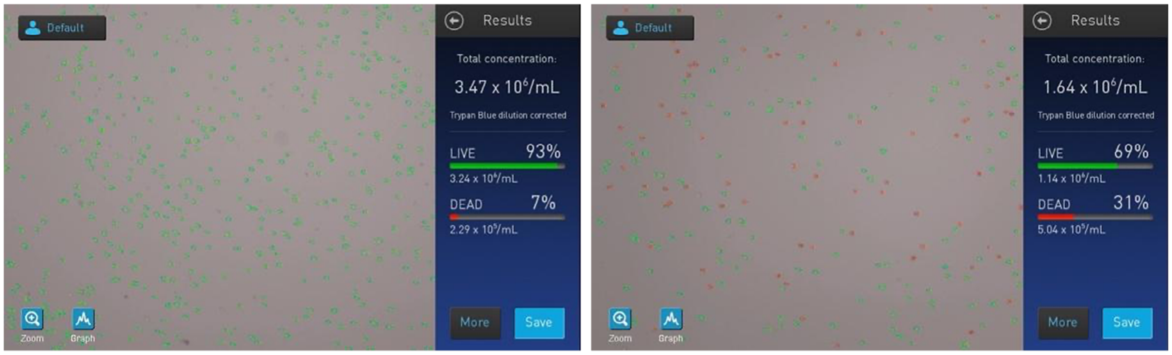

Figure 3. Representative images of high-quality cell suspension (left) and low-quality cell suspension (right).

Genomic Protocol

1. Prepare Cell Suspension

Genomic Protocol

1. Prepare Cell Suspension

This section describes the steps required to prepare a single-cell suspension, count cells, assess cell viability and cell suspension quality. The Mission Bio Tapestri workflow is optimized for a single-cell suspension input of 2,800 – 3,200 cells/µL at greater than 80% viability in a total volume of 40 µL.

NOTE

- ***Thaw reagents at room temperature unless directed to thaw them on ice.***
- ***Store reagents according to manufacturer's storage recommendations as soon as they are received. Vortex and then centrifuge reagents as directed.***
- ***The following procedure assumes cell lines, PBMCs, or BMMCs to be cryopreserved in 2 mL cryovials in a total volume of 0.5 mL and stored in liquid nitrogen or -80°C.***

1.1 Retrieve all reagents required for preparing the cell suspension:

» 1x DPBS w/o Ca^{2+}/Mg^{2+} → keep at RT

» Cell Buffer (4 °C +4 Kit) → place on ice

1.2 Remove the cryovial containing the frozen cells from liquid nitrogen storage or -80 °C freezer and **immediately place it into a 37 °C water bath**.

1.3 **Quickly thaw the cells** (< 1 minute) by gently swirling the vial in the 37 °C water bath until there is just a small bit of ice left in the vial.

1.4 **Transfer 1 mL of room temperature 1x DPBS** dropwise into the vial.

1.5 Carefully resuspend the cells by pipetting 1 time up and down and transfer the cells into a new 1.5 mL Eppendorf tube.

1.6 **Centrifuge** at appropriate speed – we suggest **300 – 400 x g for 5 minutes**. The actual centrifugation speed and duration vary from cell type to cell type. Centrifugation is complete when the pellet is visible, and the supernatant is clear.

1.7 Aspirate the supernatant without disturbing the cell pellet.

1.8 **Add 1 mL of 1x DPBS to the cell pellet** to wash the cells, then **centrifuge at 300 – 400 x g for 5 minutes**.

1.9 Aspirate the supernatant without disturbing the cell pellet.

1.10 **Add 50 µL of Cell Buffer** and carefully resuspend cells by gently pipetting up and down to disaggregate the cell pellet until no cell clumps are visible.

IMPORTANT ***Mission Bio's Cell Buffer contains density gradient medium. Cells that are resuspended in Cell Buffer are difficult to pellet via centrifugation.***

1.11 **Count the cells** using an automated cell counter and dead-cell exclusion dye (e.g., Trypan Blue) according to the manufacturer's instructions. Assess both single cell suspension quality and cell viability.

1.12 **Dilute cell suspension to ~3,000 cells/µL** using Cell Buffer in a total volume of at least 40 µL. Confirm concentration using cell counter prior to loading.

IMPORTANT ***Use of cell concentrations outside the range of 2,800 – 3,200 cells/µL or viability below 80% may adversely affect results.***

1.13 **Place cell suspension on ice** until required in ***Section 2 – Encapsulate Cells***. Do not keep cell suspensions on ice for longer than 30 minutes before proceeding to encapsulation.

Genomic Protocol

2. Encapsulate Cells

2. Encapsulate Cells

In this step, cells are encapsulated with Lysis Buffer to create a cell emulsion. For input cell concentrations of 2,800 – 3,200 cells/µL, approximately 5% of all emulsion droplets will contain a cell, following a Poisson distribution.

IMPORTANT

- ***Handle emulsions with caution, avoiding sources of static and pipetting slowly and carefully.***
- ***Use only the consumables (sample tubes and pipette tips) validated by Mission Bio (see Tapestri Instrument and DNA Cartridge and list of Required Third Party Consumable Reagents).***

2.1 Turn on the Tapestri Instrument at least 5 minutes prior to use.

2.2 Retrieve all reagents required for cell encapsulation:

- » Tapestri DNA Cartridge (Cartridge Kit) → keep at RT
- » Tapestri DNA Gasket (Cartridge Kit) → keep at RT
- » Lysis Buffer (●) (-20 °C, -20 Kit) → place on ice
- » Reverse Primer Pool (●) (-20 °C, -20 °C Oligo Pool Kit) → place on ice
- » Encapsulation Oil (4 °C, +4 Kit) → equilibrate to RT
- » Cell Suspension (prepared in ***Section 1 – Prepare Cell Suspension***) → place on ice

2.3 In a Pre-PCR area, carefully open a new Tapestri DNA Cartridge.

IMPORTANT

- ***Avoid dust and debris at all times when handling the DNA cartridge.***
- ***Each DNA cartridge is packaged with one DNA Gasket to be used throughout the run. Store both DNA cartridge and DNA Gasket in protective packaging when not in use during the experiment. Use within 24 hours after opening.***

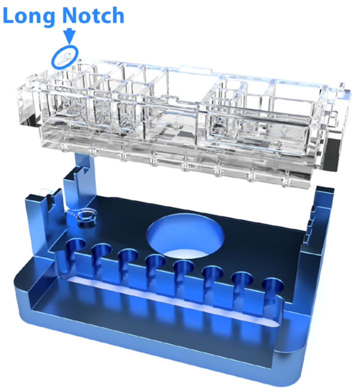

2.4 Mount the Base Plate onto the Tapestri Instrument. Pre-label and **place a 0.2 mL emulsion-safe PCR tube into the middle of the slot at the left of the Base Plate** for collecting the encapsulation emulsion product. Position the tube with the open lid facing left.

2.5 **Place the DNA Cartridge onto the Base Plate** with the long notch on the side of the cartridge oriented on the top left.

IMPORTANT ***Minimize electrostatic sources. Only Axygen MAXYmum Recovery PCR tubes (PCR-02-L-C) or (PCR-02D-L-C) and USA Scientific (1402-8120) have been validated by Mission Bio as emulsion-safe. Do not substitute with other PCR tubes.***

2.6 In a new tube, prepare **Lysis Mix** by adding **5.1 µL of Reverse Primer Pool** (●) into **65 µL of Lysis Buffer** (●), vortex and briefly centrifuge.

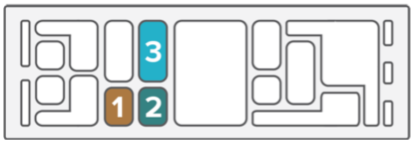

2.7 Pipette 60 µL of **Lysis Mix** into reservoir 1.

2.8 Pipette 35 µL of **Cell Suspension** into reservoir 2.

Pipette slowly into the bottom of the reservoir where the inlet is located. Raise the pipette tip as the liquid level in the reservoir is rising, keeping the tip slightly submerged.

Ensure that the inlet is fully covered with Cell Suspension before starting the Cell Encapsulation program.

2.9 Pipette 200 µL of **Encapsulation Oil** into reservoir 3. Be careful not to spill oil into surrounding reservoirs while loading the cartridge

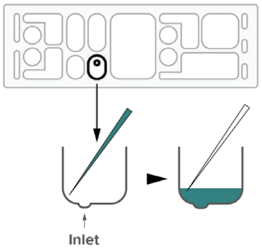

IMPORTANT ***Make sure to apply the DNA Gasket and start the program within 1 minute after loading the Encapsulation Oil.***

2.10 **Apply the Tapestri DNA Gasket** to the top of the cartridge. Ensure that it is oriented correctly.

2.11 Firmly **close the instrument lid**, until the lid handle is level and flush with the top of the lid and instrument.

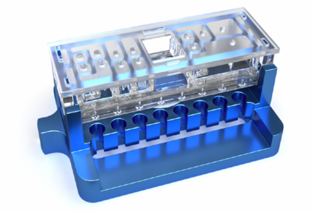

2.12 Run the **Encapsulation** program by pressing **Step 1: Encapsulation** on the Tapestri Instrument touchscreen. Press **NEXT** and confirm to start the run. The program runs for 5 minutes.

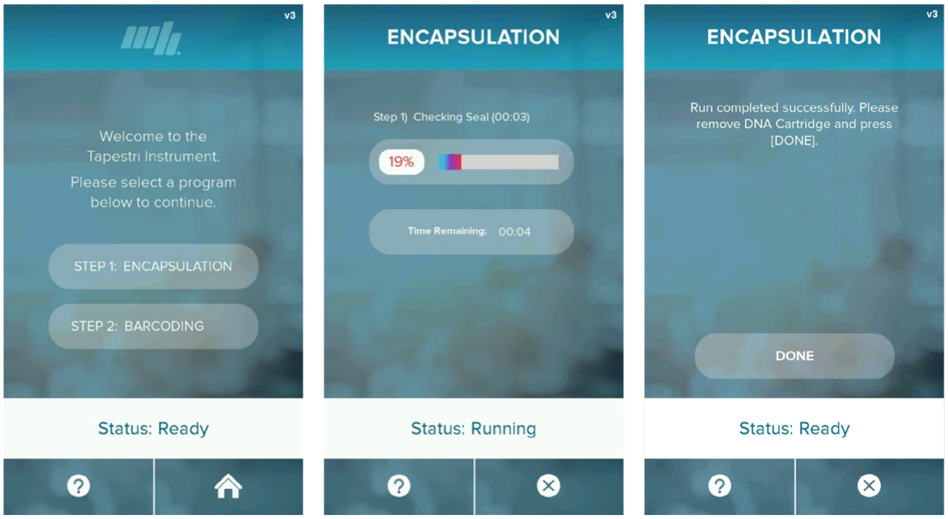

Figure 4. Touchscreen displays show main menu (left), screen after selecting 'Step 1: Encapsulation' program (middle), and final screen after Encapsulation is completed (right).

2.13 When the touchscreen displays **DONE**, carefully open the lid and **remove the cartridge and gasket from the Tapestri instrument.**

2.14 **Carefully transfer the emulsion sample tube** to a 96-well plate holder and assess emulsion quality. Encapsulated cells appear as a white layer (see Figure 5).

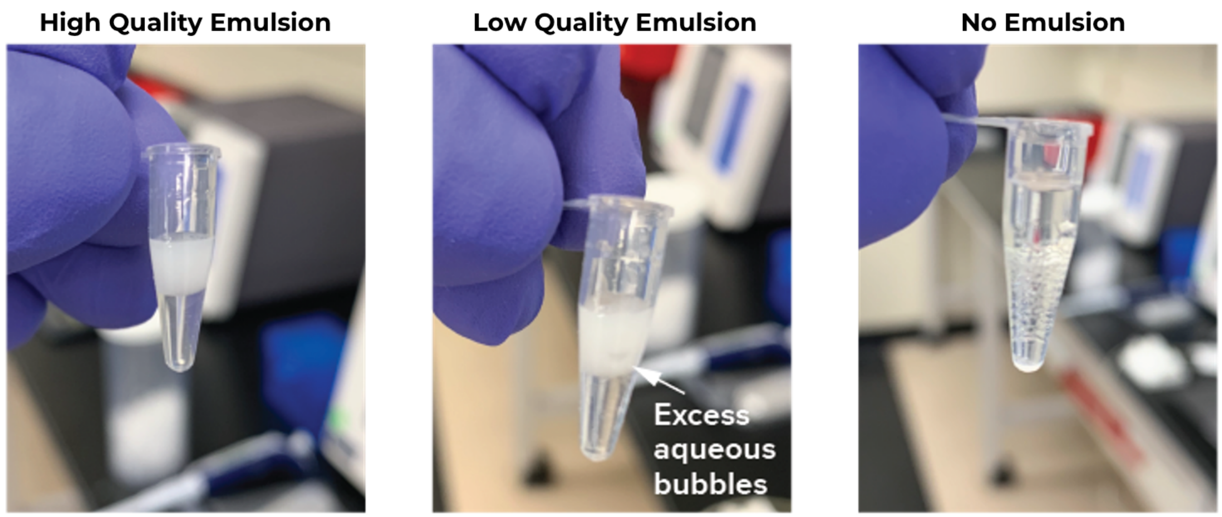

Figure 5. Emulsion Quality

If low-quality or no emulsions are detectable, please contact support@missionbio.com.

2.15 **Mount the cartridge back onto the Base Plate** seated inside the instrument and close the lid to protect it from environmental debris.

2.16 The sample tube contains 50 – 80 µL of cell emulsion (top layer) and 80 – 120 µL encapsulation oil (bottom layer) for a total volume of 130 – 200 µL.

2.17 Use a **gel loading tip** to carefully remove excess oil from the bottom layer of the tube leaving a total of **100 µL** of emulsion + oil (approximately the middle point of the tube as it narrows to the conical base).

IMPORTANT

- ***Hold the tube by the lid. Remove oil only. Make sure the gel loading tip is at the very bottom of the sample tube and wait ~5 seconds before removing oil. This will minimize removal of emulsion.***
- ***After removal, 100 µL of oil + emulsion will remain at the bottom of the tube. Make sure the entire tube volume does not exceed 100 µL.***

Genomic Protocol

3. Lyse and Digest Cells

3. Lyse and Digest Cells

In this step, cells are lysed and DNA binding proteins are enzymatically digested to make DNA accessible for downstream target amplification.

3.1 **Run the "Cell Lysis and Protein Digest"** protocol on the thermal cycler according to the manufacturer's instructions, using the following parameters:

Step	Temperature	Time
1	50 °C	60 min
2	80 °C	10 min
3	4 °C	HOLD

Table 1. Thermal cycling protocol for 'Lysis/Digest'.

3.2 When the run completes, store the lysed and digested samples at 4 °C until required in ***Section 4 – Barcode Cells***. The volume of oil at the bottom of the tube is expected to increase slightly after thermal cycling.

NOTE ***We strongly recommend proceeding through Section 4 – Barcode Cells on day 1. If necessary, the encapsulation emulsion products may be stored at 4 °C overnight, upright in a sealed container to avoid condensation.***

Genomic Protocol

4. Barcode Cells

4. Barcode Cells

In this step, the droplets containing encapsulated cell lysate are combined with drops containing both Barcoding Mix and Barcoding Beads. These newly generated drops are then distributed into 8 PCR collection tubes, to create 8 cell-barcoding emulsion samples.

4.1 Retrieve all reagents required for Cell Barcoding:

- » Barcoding Mix (-20 °C, -20 Kit) → place on ice
- » Barcoding Beads (●) (-20 °C, -20 Barcoding Bead Kit) → *thaw at room temperature and protect from light*
- » Forward Primer Pool (●) (-20 °C, -20 Oligo Pool Kit) → place on ice
- » Barcoding Oil (4 °C, +4 Kit) → equilibrate to RT
- » Electrode Solution (4 °C, +4 Kit) → equilibrate to RT

IMPORTANT ***Protect Barcoding Beads from light and thaw at room temperature.***

Prime the DNA Cartridge for Barcoding

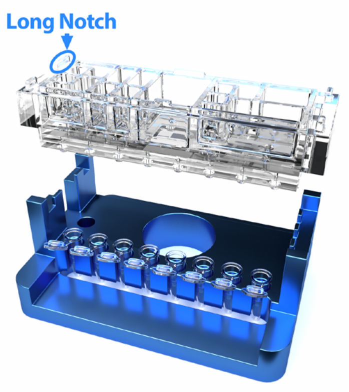

IMPORTANT ***Use emulsion-safe PCR tubes.***

4.2 Place eight 0.2 mL emulsion-safe PCR tubes **into the eight slots at the bottom of the Tapestri Base Plate** with the open lids toward you.

4.3 Mount the Tapestri DNA Cartridge (used during Cell Encapsulation) onto the Base Plate.

4.4 Pipette **200 µL** of **Electrode Solution** into each reservoir 4 of the cartridge.

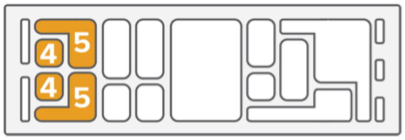

4.5 Pipette **500 µL** of **Electrode Solution** into each reservoir 5 of the cartridge.

4.6 **Apply the DNA Gasket** and firmly close the instrument lid, until the lid handle is level and flush with the top of the lid and instrument.

4.7 Run the **Priming** program by pressing **Step 2: Barcoding** on the Tapestri Instrument touchscreen. Press **NEXT** and confirm to start the program. The **program runs for 20 minutes** before automatically pausing to allow for loading of the remaining reagents.

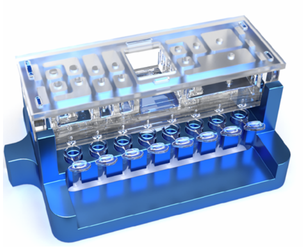

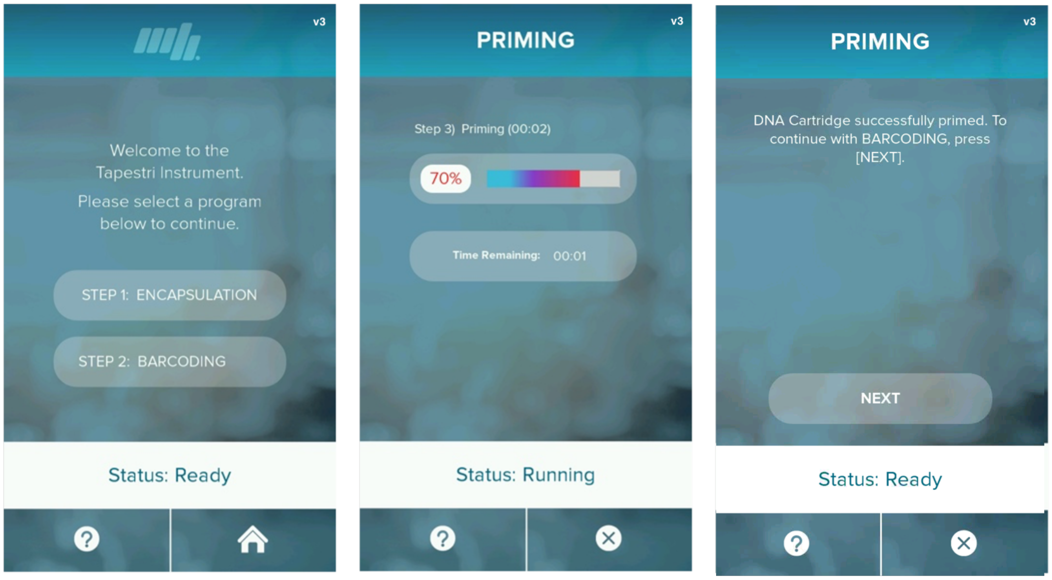

Figure 6. Touchscreen displays show main menu (left), screen after selecting 'Step 2: Barcoding'. program (middle), and final screen after Priming is completed (right).

Prepare Barcoding Mix

4.8 **Prepare 300 µL Barcode Mix** as shown in the following table. A total of 267 µL will be used for Barcoding.

Reagent	Volume (µL)
Barcoding Mix	295
Forward Primer Pool (⚪)	5
**Total**	**300**

Table 2. Reagents for Barcode Mix.

4.9 **Briefly vortex the Barcode Mix** and centrifuge to collect the contents and store on ice.

IMPORTANT ***After the Priming program has completed the Barcoding program must be started within 30 minutes.***

Load the DNA Cartridge

4.10 Once priming is complete and the instrument screen displays "NEXT", quick-spin Barcoding Beads (🔵) to collect contents.

4.11 Take **67 µL** of the prepared Barcode Mix and add it to the bead tube.

IMPORTANT ***Remember to avoid sources of static and pipette slowly and carefully when handling emulsions.***

4.12 **Retrieve the emulsion containing the encapsulated cell lysate** from the thermal cycler at 4 °C (see ***Section 3 – Lyse and Digest Cells***).

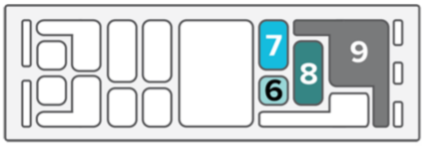

4.13 Open the instrument lid and **slowly pipette all of the contents of the encapsulated emulsion** into **reservoir 6**.

4.14 *Vortex the Barcoding Beads at full speed for 1 min.* Carefully pipette **250 µL of Barcoding Beads** (●) **into reservoir 7**. *Pipette slowly and do not introduce bubbles.*

4.15 Pipette **200 µL of Barcoding Mix** into **reservoir 8**.

4.16 Pipette **1.25 mL of Barcoding Oil** into **reservoir 9**. Be careful not to spill oil into surrounding reservoirs while loading the cartridge.

IMPORTANT ***Make sure to apply the DNA Gasket and start the Barcoding program within 1 minute of loading the Barcoding Oil.***

4.17 **Apply the DNA Gasket** and firmly close the instrument lid, until the lid handle is level and flush with the top of the lid and instrument.

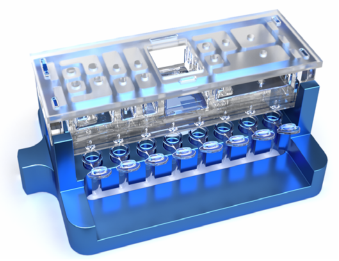

4.18 **Run the Barcoding program** by pressing NEXT on the Tapestri Instrument touchscreen in the following figure. This program will **complete in 45 minutes**.

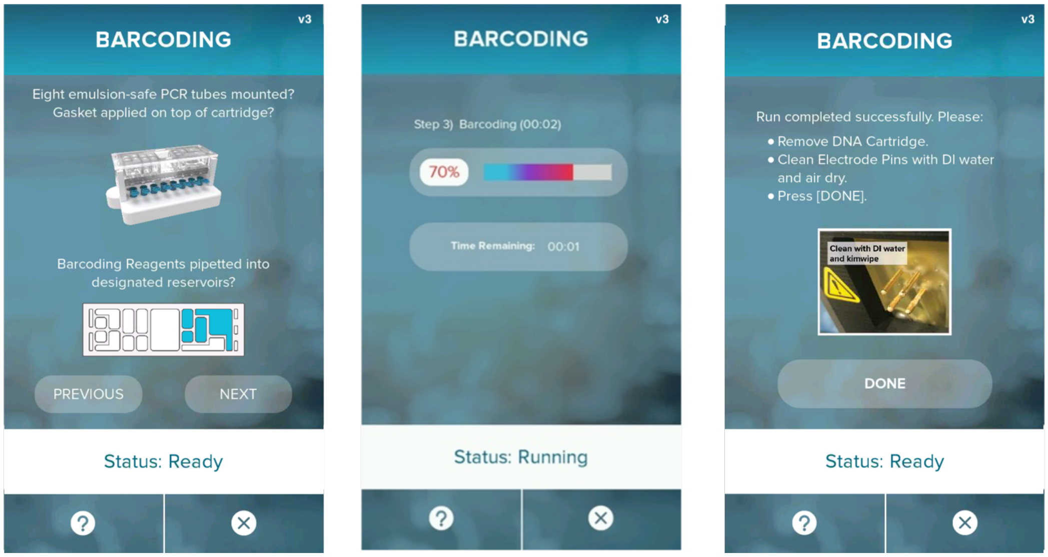

Figure 7. Touchscreen displays before the second part of Barcoding (left), the status during Barcoding (middle), and final screen after Barcoding is completed (right).

4.19 When the screen displays **Run completed successfully**, press DONE, carefully open the lid and **remove the Base Plate together with the cartridge to collect the eight tubes** containing the barcoded emulsion.

NOTE ***The volumes of oil and emulsion may vary across all 8 tubes. Occasionally, a tube may be empty; this is not cause for concern as all 8 channels are connected. If more than 190 μL of Barcoding Beads or more than 15 μL of emulsions remain in reservoirs proceed with the workflow and contact support@missionbio.com.***

4.20 Visually evaluate the emulsion quality. The barcoded **emulsions are visible as a white layer** on top of the oil layer.

4.21 Use a **gel loading tip** to carefully remove excess oil from the bottom layer of all eight tubes, leaving a **total of 100 μL of emulsion + oil** (approximately the middle point of the tube as it narrows to the conical base) per tube.

IMPORTANT ***Hold tubes by the lid. Insert pipette tip only once when slowly removing oil.***

Clean Electrode Pins

NOTE ***The electrode pins on the bottom of the instrument lid are in direct contact with the Electrode Solution during Priming and Cell Barcoding. Gradual buildup of salt deposits might eventually hinder instrument function. Electrodes are disabled when the instrument lid is open.***

4.22 With a dust-free cloth and deionized water clean all four electrode pins on the bottom of the instrument lid.

4.23 Dry the electrode pins using a dry dust-free cloth.

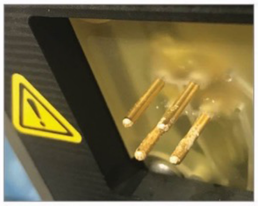

Dirty (salt deposits)

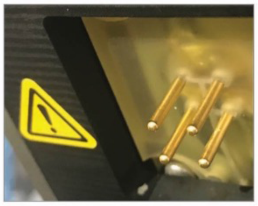

Clean

Genomic Protocol

5. Targeted PCR Amplification

5. Targeted PCR Amplification

5.1 **Transfer the samples to a thermal cycler, and run the "Targeted PCR" protocol** according to the manufacturer's instructions.

Make sure to select the correct thermal cycling program with the **correct annealing/ extension times (Steps 4 and 8, see Table 4 below)** that are compatible with the targeted DNA panel you processed your samples with.

IMPORTANT ***Ensure that the emulsions in all eight tubes (white top layer) sit within the height of the block of the thermal cycler that is temperature controlled. Use a PCR skirt to ensure even heat transfer.***

		Amplicon Number	20 – 100	101 – 200	201 – 300	> 300	
**Step**	**Ramp**	**Temperature**	**Time**	**Time**	**Time**	**Time**	**Cycle**
1	4 °C/s	98 °C	6 min	6 min	6 min	6 min	
2	1 °C/s	95 °C	30 sec	30 sec	30 sec	30 sec	10
3		72 °C	10 sec	10 sec	10 sec	10 sec	
4		61 °C	**3 min**	**4.5 min**	**6 min**	**9 min**	
5		72 °C	20 sec	20 sec	20 sec	20 sec	
6	1 °C/s	95 °C	30 sec	30 sec	30 sec	30 sec	10
7		72 °C	10 sec	10 sec	10 sec	10 sec	
8		48 °C	**3 min**	**4.5 min**	**6 min**	**9 min**	
9		72 °C	20 sec	20 sec	20 sec	20 sec	
10	4 °C/s	72 °C	2 min	2 min	2 min	2 min	
11		4 °C	HOLD	HOLD	HOLD	HOLD	

Table 3. Thermal cycling programs for Targeted PCR.

IMPORTANT ***Ensure ramp rate is set to 1 °C/s for emulsion stability. If you observe an aqueous layer on top of the white-appearing emulsion layer, please contact support@missionbio.com.***

Emulsions not intact
Targeted PCR unsuccessful

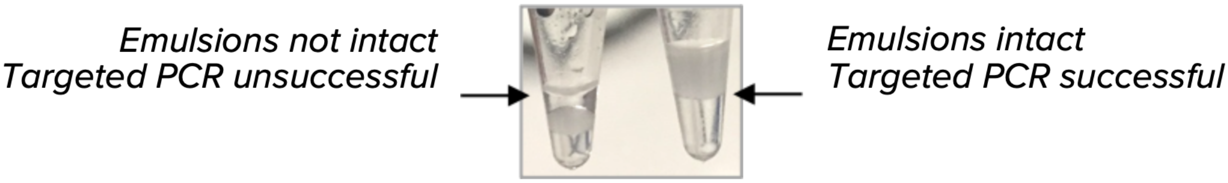

Emulsions intact
Targeted PCR successful

NOTE ***STOPPING POINT: Emulsions can be left on thermocycler at 4 °C overnight.***

Break Emulsions and Pool Tubes

5.2 Retrieve the following reagents needed for PCR product purification:

» Extraction Agent (●) (+4 °C, +4 Kit) → equilibrate to RT

» Nuclease-free water

5.3 **Add 10 µL of Extraction Agent (●) to each sample tube.** Vortex briefly and spin for 20 seconds.

5.4 **Incubate at room temperature** for 3 minutes until the entire emulsion changes from white to clear in color. *If emulsions don't lose their white appearance, add 5 µL of additional Extraction Agent, vortex briefly and spin for 30 more seconds.*

5.5 **Add 45 µL of nuclease-free water to each of the tubes.** Mix by briefly vortexing and then spinning for 10 seconds in a benchtop centrifuge to separate the aqueous and oil layers.

5.6 Pipette **42 µL of the aqueous top layer** from each tube into one **new 1.5 mL DNA LoBind Eppendorf tube. Pool contents from tubes 1 – 8**. Total volume will be 336 µL. *Do not transfer any oil or Barcoding Beads.*

5.7 Store sample at 4 °C or proceed to ***Section 6 – Cleanup PCR Products***.

NOTE ***STOPPING POINT: This is a good place to stop in the protocol if there is not adequate time to continue to clean up the libraries in one day (~ 2.5 hr). The amplified PCR products can be stored at 4 °C for < 24 hours or -20 °C for > 24 hours.***

Genomic Protocol

6. Cleanup PCR Products

6. Cleanup PCR Products

Digest PCR Product

6.1 Retrieve all reagents required for digesting the PCR product:

- » DNA Clean up Buffer (●) (-20 °C, -20 Kit) → place on ice
- » Clean up Enzyme (●) (-20 °C, -20 Kit) → place on ice

6.2 To the pooled sample (336 µL), add **40 µL DNA Clean up Buffer** (●) and **24 µL Clean up Enzyme** (●). Total volume will now be 400 µL.

6.3 Briefly vortex and quick-spin the tube.

6.4 Transfer the tube to a thermo mixer and **digest at 37 °C for 60 minutes**.

6.5 While sample is digesting, equilibrate AMPure XP to room temperature.

6.6 Remove the tube from the thermo mixer, store at room temperature and continue with AMPure XP Library Cleanup.

AMPure XP Library Cleanup

6.7 Thoroughly **vortex AMPure XP reagent for 45 seconds** at high-speed. Equilibrate the **AMPure XP reagent to room temperature.**

6.8 Prepare **5 mL fresh 80% ethanol** using nuclease-free water.

NOTE ***Measure volumes for 100% ethanol and nuclease-free water separately. Make sure to tightly close all ethanol containers when not in use, since ethanol can absorb water over time, leading to lower concentrations.***

6.9 **Spin down sample tube for 20 seconds. If a pellet is visible, transfer clear aqueous solution to a new tube, being careful not to disturb the pellet. Add nuclease-free water to achieve a total volume of 400 µL.**

6.10 Add **400 µL of nuclease-free water** to tube (total volume = 800 µL). Quantify volume using a pipette; if necessary, add more water to adjust volume to 800 µL.

6.11 Thoroughly vortex AMPure XP reagent at high speed immediately prior to usage.

6.12 Add **576 µL (0.72X)** of AMPure XP reagent to tube. Vortex for **5 seconds** and quick-spin to collect contents.

6.13 Incubate the tube at **room temperature for 5 minutes**.

6.14 Place on magnet and wait **5 minutes** for the AMPure XP beads to separate from solution.

6.15 Without removing the the tube from the magnet, **remove the clear liquid from the tube** and discard.

6.16 **Wash AMPure XP bead pellet** while keeping the tube on the magnet:

- a. Carefully **add 1 mL** of the freshly prepared **80% ethanol.**
- b. Wait **30 seconds.**
- c. **Remove ethanol** without disturbing the AMPure XP beads.
- d. **Repeat** steps a – c once, for a total of two wash cycles.

6.17 Keeping the tube on the magnet, using a P-10 pipette, **remove all residual ethanol** from the tube without disturbing the AMPure XP beads.

6.18 **Dry AMPure XP bead pellet** in the tube on the magnet by incubating at room temperature for **5 minutes**. *Over-dried beads may be more difficult to suspend.*

6.19 Remove the tube from the magnet.

6.20 **Add 110 µL** of nuclease-free water into the tube.

6.21 **Vortex tube for 10 seconds**, quick-spin to collect the contents, and incubate the tube at room temperature for **2 minutes**.

6.22 Place the tube onto the magnet and **wait for at least 2 minutes** or until solutions are clear.

6.23 **Transfer 100 µL** of purified PCR product from the tube to a new 0.2 mL PCR tube. *Avoid transfer of AMPure XP beads.*

6.24 Thoroughly **vortex AMPure XP reagent.** Add **76 µL (0.76X)** of AMPure XP reagent to the tube with eluted PCR product (176 µL total). Vortex for 5 seconds and quick-spin to collect the contents.

6.25 Incubate the tube at room temperature for **5 minutes**.

6.26 Place the tube onto the magnet, wait **5 minutes** for the beads to separate from the solution.

6.27 Without removing the tube from the magnet, remove the supernatant and discard.

6.28 **Wash AMPure XP bead pellet** while keeping the tube on the magnet:

- a. Carefully **add 200 µL** of the freshly prepared **80% ethanol.**
- b. Wait **30 seconds.**
- c. **Remove ethanol** without disturbing the AMPure XP beads.
- d. **Repeat** steps a - c once, for a total of two wash cycles.

6.29 Keeping the tube on the magnet, using a P-10 pipette, **remove all residual ethanol** from the tube without disturbing the AMPure XP beads.

6.30 **Dry AMPure XP bead pellet** in the tube on the magnet by incubating at room temperature for **5 minutes**. *Over-dried beads may be more difficult to suspend.*

6.31 Remove the tube from the magnet.

6.32 **Add 110 µL** of nuclease-free water into the tube.

6.33 **Vortex tube for 10 seconds**, quick-spin to collect the contents, and incubate the tube at room temperature for **2 minutes**.

6.34 Place the tube onto the magnet and **wait for at least 2 minutes** or until solutions are clear.

6.35 **Transfer 100 µL** of purified PCR product from the tube to a new 0.2 mL PCR tube. *Avoid transfer of AMPure XP beads.*

6.36 Store the purified PCR product on ice and proceed to the next step, or store at -20 °C long term.

NOTE ***STOPPING POINT: This is a good place to stop in the protocol if there is not adequate time to continue to Library PCR (~ 1 hr). The purified PCR products must be stored at 4 °C or -20 °C and will be stable for up to six months.***

Genomic Protocol

7. Library PCR

7. Library PCR

During Library PCR the P5 and P7 adapter sequences (Illumina) are added to the DNA product for sequencing. Each Libray Index includes both an i5 and i7 index adapter.

Use the following index combinations when indexing your samples.

# of Samples	Recommended Indices
1	2
2	2 + 3
3	2 + 3 + 4
4	2 + 3 + 4 + 7
5	2 + 3 + 4 + 7 + 5
6	2 + 3 + 4 + 7 + 5 + 6
7	2 + 3 + 4 + 7 + 5 + 6 + 1
8	2 + 3 + 4 + 7 + 5 + 6 + 1 + 8

Table 4. Index combinations for different sample multiplexing schemes.

7.1 Retrieve the following reagents required for Library PCR

- Purified PCR products (from the previous step)
- Library Indices 1 – 8 (●) (-20 °C, -20 Kit) → place on ice
- Library Mix (●) (-20 °C, -20 Kit) → place on ice

7.2 In a Pre-PCR area **label one new 0.2 mL PCR tube** with the index number of the Library Indices and **add 15 µL of undiluted Targeted DNA PCR product from Step 6.35** in ***Chapter 6 – Cleanup PCR Products***. The remaining stock solutions of purified PCR products at -20 °C.

7.3 **Add 25 µL of Library Mix** (●) **and 10 µL of Library Indices** (●) to the tube containing 15 µL of sample for a total of 50 µL.

IMPORTANT ***Make sure to avoid cross-contamination when handling the indices.***

7.4 **Vortex and quick-spin** the tube to collect contents.

7.5 Transfer the sample to the thermal cycler, then run the Library PCR protocol according to the manufacturer's instructions, using the following parameters on the following page:

<table>
<tr><th>Step</th><th>Temperature</th><th>Time</th><th>Cycle</th></tr>
<tr><td>1</td><td>95 °C</td><td>3 min</td><td></td></tr>
<tr><td>2</td><td>98 °C</td><td>20 sec</td><td rowspan="3">10</td></tr>
<tr><td>3</td><td>62 °C</td><td>20 sec</td></tr>
<tr><td>4</td><td>72 °C</td><td>45 sec</td></tr>
<tr><td>5</td><td>72 °C</td><td>2 min</td><td></td></tr>
<tr><td>6</td><td>4 °C</td><td>HOLD</td><td></td></tr>
</table>

Table 5. Thermal cycling program for Library PCR.

7.6 Remove the sample from the thermal cycler and store at room temperature.

Library cleanup

NOTE ***Always use freshly prepared 80% ethanol.***

7.7 Thoroughly **vortex AMPure XP reagent for 45 seconds** at high-speed. Equilibrate the **AMPure XP reagent to room temperature.**

7.8 Thoroughly **vortex AMPure XP reagent** at high-speed immediately prior to usage.

7.9 Add **50 µL of nuclease-free water to the sample tube.**

7.10 **Add 69 µL (0.69X) of AMPure XP reagent** to the 100 µL sample.

7.11 **Vortex for 10 seconds** and quick-spin to collect contents.

7.12 Incubate the tube at **room temperature for 5 minutes,** and then place the tube on the magnet.

7.13 Allow at least **2 minutes** for the AMPure XP beads to separate from solution.

7.14 Without removing the tube from the magnet, **remove the supernatant** and discard. *The DNA is adhered to the beads.*

7.15 **Wash AMPure XP bead pellets** while keeping the tube on the magnet:

- » a. Carefully **add 200 µL** of the freshly prepared **80% ethanol.**
- » b. Wait **30 seconds.**
- » c. **Remove ethanol** without disturbing the AMPure XP beads.
- » d. **Repeat** steps a – c once, for a total of two wash cycles.

7.16 Keeping the tube on the magnet, **remove all residual ethanol** without disturbing the AMPure XP beads.

7.17 **Dry AMPure XP bead pellets** in the tube on the magnet by incubating at room temperature for at least **2 minutes.** *Over-dried beads may be more difficult to suspend.*

7.18 Remove the tube from the magnet.

7.19 **Add 110 µL** of nuclease-free water into the tube.

7.20 **Vortex tube for 5 seconds,** quick-spin to collect the contents, and incubate at room temperature for **2 minutes.**

7.21 Place the tube onto the magnet and **wait for at least 2 minutes** or until solutions are clear.

7.22 Transfer 100 µL of purified PCR product from the tube to a new 0.2 mL PCR tube. *Avoid transfer of AMPure XP beads.*

7.23 Thoroughly **vortex AMPure XP reagent. Add 72 µL (0.72X) of AMPure XP reagent** to the 100 µL sample.

7.24 **Vortex for 5 seconds** and quick-spin to collect contents.

7.25 **Incubate the tube at room temperature** for 5 minutes, and then place the tube on the magnet.

7.26 Allow at least **2 minutes** for the AMPure XP beads to separate from solution.

7.27 Without removing the tube from the magnet, **remove the supernatant** and discard. *The DNA is bound to the beads.*

7.28 **Wash AMPure XP bead pellets** while keeping the tube on the magnet:

- » a. Carefully **add 200 µL** of the freshly prepared 80% ethanol.
- » b. **Wait 30 seconds.**
- » c. **Remove ethanol** without disturbing the AMPure XP beads.
- » d. **Repeat** steps a – c once, for a total of two wash cycles.

7.29 Keeping the tube on the magnet, **remove all residual ethanol** without disturbing the AMPure XP beads.

7.30 **Dry AMPure XP bead pellets** in the tube on the magnet by incubating at room temperature for at least 2 minutes. *Over-dried beads may be more difficult to suspend.*

7.31 Remove the tube from the magnet.

7.32 **Add 15 µL of nuclease-free water** into the tube.

7.33 **Vortex tube for 5 seconds**, quick-spin to collect the contents, and incubate at room temperature for 2 minutes.

7.34 **Place the tube onto the magnet and wait** for at least 2 minutes or until solutions are clear.

7.35 **Transfer 12 µL of purified PCR product** from the tube to a new 0.2 mL PCR tube. *Avoid transfer of AMPure XP beads.*

7.36 Store purified DNA library at -20 °C until proceeding to the next step.

NOTE ***STOPPING POINT: This is a good place to stop in the protocol if there is not adequate time to finish in one day (~ 1 hr). The purified Library PCR products can be stored at -20 °C.***

Genomic Protocol

8. Quantify and Normalize Sequencing Library

8. Quantify and Normalize Sequencing Library

8.1 Retrieve the following for library quantitation:

- Purified sample libraries
- Agilent DNA High Sensitivity Kit or Agilent DNA 1000 kit
- Qubit™ dsDNA HS Kit

Quantify Libraries

NOTE ***Agilent TapeStation 2200/4200 or Fragment Analyzer (Advanced Analytical) may be used if an Agilent Bioanalyzer 2100 is not available.***

8.2 Follow Qubit protocol to verify library concentration.

8.3 Verify the DNA Library product size and purity and quantify following manufacturer's instructions.

NOTE ***A final concentration of on-target product > 2 ng/µL can be expected.***

Library Size Distribution

Libraries generated with catalog panels in general produce high-quality on-target amplicons with only a few off-target fragments (e.g., primer dimers).

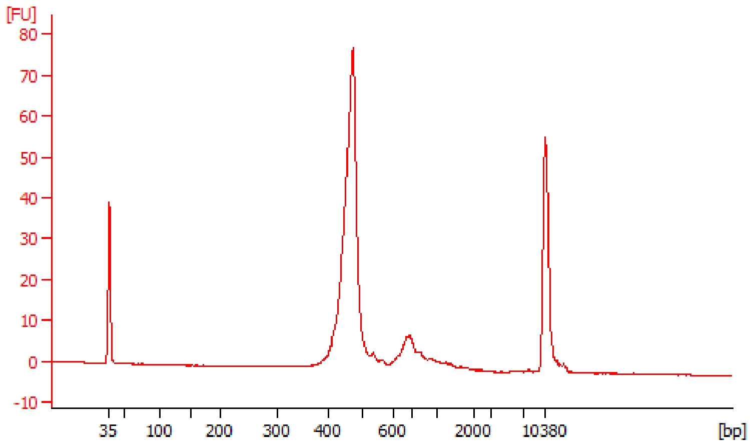

8.4 Quantify the concentration of the libraries based on a range of 100 – 700 bp to include products that may efficiently cluster on the Illumina flow cell. This minimizes the potential to over-cluster when sequencing the libraries. *Use this value in Step 8.5.*

NOTE ***If significant quantities (> 5%) of smaller products are seen at < 300 bp (e.g., primer dimers), contact support@missionbio.com for additional support. An additional AMPure cleanup step may be required.***

Normalize and Pool Libraries

8.5 Use the ***Library Quantification and Pooling Tool*** to dilute each tube library.

8.6 **Re-quantify the pooled library** with a Qubit Fluorometer.

NOTE ***Alternatively pooled libraries may be quantified using quantitative PCR (KAPA Library Quantification Kit Illumina Platforms, PN KK4873).***

Genomic Protocol

9. Sequence Library

9. Sequence Library

Parameter	Specification
Final library size	**350 bp – 550 bp** with peak at ~460 bp
Supported sequencers	MiSeq, HiSeq 2500, NextSeq 1000/2000, NextSeq 550, HiSeq 3000/4000, NovaSeq 5000/6000, NovaSeq X
Index 1 (i7)	**Yes** (8nt). Index 1 – 8 sequences are different from Illumina's index sequences.
Index 2 (i5)	**Yes** (8nt). Index 1 – 8 sequences are different from Illumina's index sequences.
Number of unique i7/i5 index pair per sample	**1**
Custom sequencing primer?	**No**
Sequencing chemistry	**2 x 150 bp** recommended. **2 x 250** supported for DNA only.
PhiX %	**5 % – 20 %** see *Library Quantification and Pooling Tool*
Compatible with non-Tapestri libraries?	**Yes,** if libraries are of similar size.
Number of expected FASTQ files per sample	**2: one Read 1/Read 2 pair representing one unique i7/i5 combination**.

Table 6. Sequencing Specifications

NOTE

- ***For expanded indexing options, refer to the Mission Bio Support Page Additional Indexes Guide - DNA Libraries***
- ***For sequencing guidance, refer to the Mission Bio Support Page Library Quantification and Pooling Tool***

Sequence Information for Library Indices 1 – 8

Index	Sequence i7	Sequence i5	Reverse Sequence i5
1	CTGATCGT	ATATGCGC	GCGCATAT
2	ACTCTCGA	TGGTACAG	CTGTACCA
3	TGAGCTAG	AACCGTTC	GAACGGTT
4	GAGACGAT	TAACCGGT	ACCGGTTA
5	CTTGTCGA	GAACATCG	CGATGTTC
6	TTCCAAGG	CCTTGTAG	CTACAAGC
7	CGCATGAT	TCAGGCTT	AAGCCTGA
8	ACGGAACA	GTTCTCGT	ACGAGAAC

Table 7. Sequence nucleotide information for Library Indices 1 – 8.

Cartridge Map

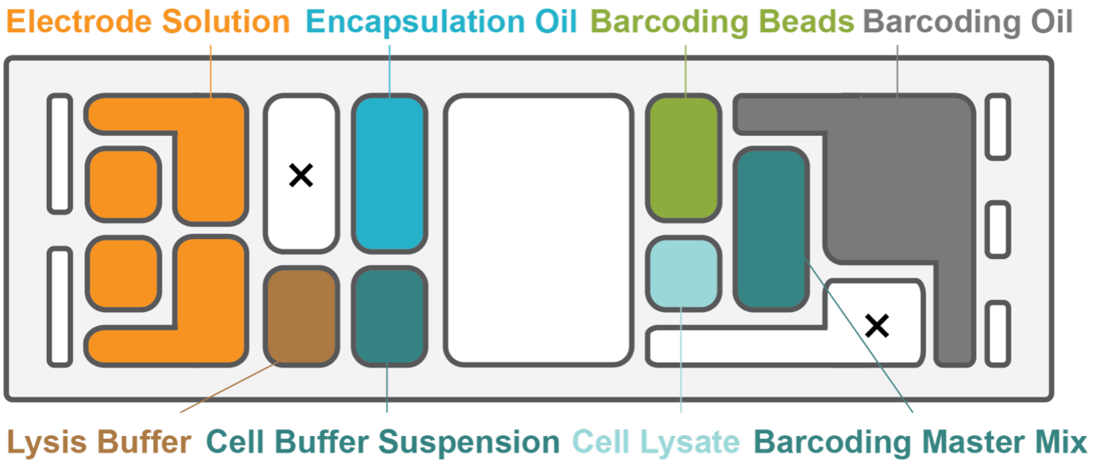

Kit Contents

TAPESTRI SINGLE-CELL DNA CORE -20 KIT v3

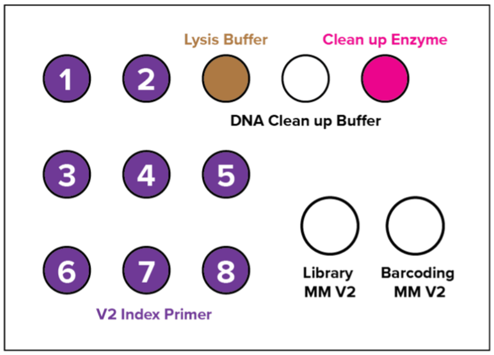

TAPESTRI SINGLE-CELL DNA CORE +4 KIT v3

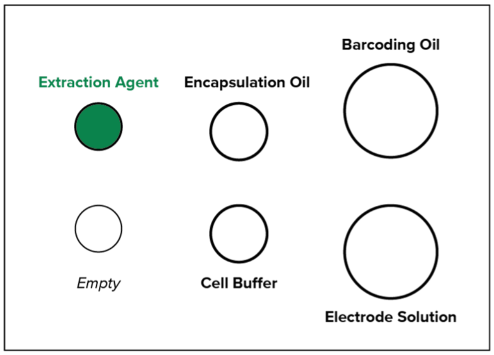

TAPESTRI SINGLE-CELL DNA BEAD KIT v3

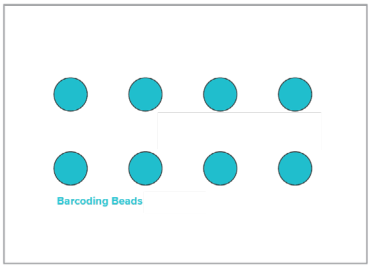

TAPESTRI SINGLE-CELL DNA CARTRIDGE KIT v3

2X

4x Cartridges

4x Gaskets

TAPESTRI SINGLE-CELL DNA OLIGO POOL

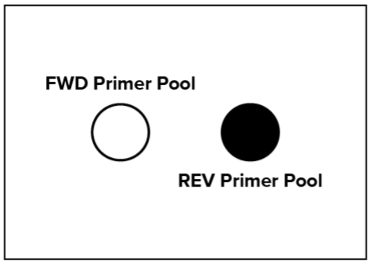

Tapestri Instrument Specifications

- Model: Tapestri Instrument
- Part Number: MB01-0020
- Mains Voltage: 115 VAC
- Frequency: 50/60 Hz
- Current: 1.0 A Max.
- Circuit Breaker: 16 Amp
- Ambient Temperature Range: 15 °C to 30 °C (59 °F – 86 °F)
- Relative Humidity (Non-Condensing): 5% to 85%
- Maximum Altitude: 6,562 ft (2,000 m)
- HV Cable Length: 24” (1500 mm)
- Overall Dimensions. H/W/D: 10.6”/27 cm x 13.7”/35 cm x 13.2”/33.6 cm

References

1. Lgr6 is a stem cell marker in mouse skin squamous cell carcinoma. P.Y. Huang et al., Nature Genetics 49(11):1624-1632 (2017).

2. RNA-Seq following PCR-based sorting reveals rare cell transcriptional signatures. M. Pellegrino, A. Sciambi, J.L. Yates, J. Mast, C. Silver, D.J. Eastburn, BMC Genomics 17:361 (2016).

3. Ultrahigh-Throughput Mammalian Single-Cell Reverse-Transcriptase Polymerase Chain Reaction in Microfluidic Drops. D.J. Eastburn, A. Sciambi, A.R. Abate, Analytical Chemistry 85, 8016 (2013).

4. Microfluidic droplet enrichment for targeted sequencing. D.J. Eastburn, Y. Huang, M. Pellegrino, A. Sciambi, L. Ptáček, A. Abate, Nucleic Acids Research Jul 27; 43(13):e86. (2015).

5. Picoinjection enables digital detection of RNA with droplet rt-PCR. D.J. Eastburn, A. Sciambi, A.R. Abate, PLoS ONE 8(4): e62961 (2013).

6. Identification and genetic analysis of cancer cells with PCR-activated cell sorting. D.J. Eastburn, A. Sciambi, A.R. Abate, Nucleic Acids Research 42, e128 (2014).

mission bio

Visit www.missionbio.com for additional support.
